# Swelling and Viscoelastic Properties of Cellulose-Based Hydrogels Prepared by Free Radical Polymerization of Dimethylaminoethyl Methacrylate in Cellulose Solution

**DOI:** 10.3390/gels9020094

**Published:** 2023-01-21

**Authors:** Roko Blažic, Katarina Marušić, Elvira Vidović

**Affiliations:** 1 Faculty of Chemical Engineering and Technology, University of Zagreb, Marulićev trg 19, HR-10000 Zagreb, Croatia; 2 Radiation Chemistry and Dosimetry Laboratory, Division of Materials Chemistry, Ruđer Bošković Institute, Bijenička cesta 54, HR-10000 Zagreb, Croatia

**Keywords:** swelling properties, viscoelastic properties, ionizing radiation, silver particles, Fe^3+^ adsorption

## Abstract

The grafting of a stimuli-responsive polymer (poly(dimethylaminoethyl methacrylate)) onto cellulose was achieved by performing free radical polymerization of a vinyl/divinyl monomer in cellulose solution. The grafting and crosslinking efficiency in the material have been increased by subsequent irradiation of the samples with ionizing radiation (doses of 10, 30, or 100 kGy). The relative amount of poly(dimethylaminoethyl methacrylate) in the prepared hydrogels was determined by infrared spectroscopy. The swelling behavior of the hydrogels was studied thoroughly, including microgelation extent, equilibrium swelling, and reswelling degree, as well as the dependence on the gelation procedure. The dynamic viscoelastic behavior of prepared hydrogels was also studied. The tan *δ* values indicate a solid-like behavior while the obtained hydrogels have a complex modulus in the range of 14–39 kPa, which is suitable for hydrogels used in biomedical applications. In addition, the incorporation of Ag particles and the adsorption of Fe^3+^ ions were tested to evaluate the additional functionalities of the prepared hydrogels. It was found that the introduction of PDMAEMA to the hydrogels enhanced their ability to synthesize Ag particles and absorb Fe^3+^ ions, providing a platform for the potential preparation of hydrogels for the treatment of wounds.

## 1. Introduction

Poly(dimethylaminoethyl methacrylate) (PDMAEMA) as a pH- and thermo-responsive polymer is widely researched in the field of stimuli-responsive materials, especially stimuli-responsive hydrogels because PDMAEMA-based materials display a potential for applications in diverse areas, such as antibacterial materials, smart drug delivery systems, membranes, sensors, paints, etc. [1,2,3]. The tertiary amino groups in PDMAEMA are protonated under acidic conditions, which makes their swelling dependent on pH [1]. In addition, PDMAEMA is one of the polymers that exhibit antimicrobial activities against yeast, gram-positive bacteria, gram-negative bacteria, and viruses [4]. Therefore, it is often used when antimicrobial properties are required. The presence of amine groups in PDMAEMA opens the possibility of using PDMAEMA as a reducing agent in the synthesis of silver nanoparticles [5,6].

Polysaccharide hydrogels are materials with a high water content that are being intensively researched for use in tissue engineering, drug delivery, and various sensory applications [7]. The development of hydrogels from cellulose, a naturally occurring polysaccharide with biocompatible and biodegradable properties, is of great importance. Moreover, the preparation of cellulose-based hydrogels with high water content and mechanical properties comparable to those of tendons is made possible by the intra- and intermolecular hydrogen bonds present in cellulose [8]. However, intra- and intermolecular hydrogen bonds limit the solubility of neat cellulose to specific solvents, such as *N,N*-dimethylacetamide (DMAc)/LiCl. The poor solubility and non-meltable nature of cellulose limit its processability and possible application for a myriad of advanced materials, while modification of cellulose enriches cellulose-based materials with new functionalities and broadens their possible applications [9,10,11]. For example, by combining PDMAEMA with cellulose, a range of biodegradable and biocompatible materials can be produced. The related literature shows that PDMAEMA grafting onto cellulose was primarily performed on cellulose derivatives such as hydroxypropylcellulose [1], carboxymethyl cellulose (CMC) [12,13], and ethyl cellulose [14]. Cellulose derivatives are further modified either by radical polymerization in their solution or by preparing a macroinitiator. Ma et al. grafted PDMAEMA onto the cellulose derivative hydroxypropyl cellulose in two steps [1]. In the first step, a macroinitiator with the desired degree of substitution of the bromoisobutyryl group was prepared, and then polymerization was carried out in a methanol–water mixture. Salama et al. used the free radical copolymerization of DMAEMA and *N*,*N*-methylenebis(acrylamide) (MBA) in an aqueous solution of cellulose derivative CMC in order to modify it [12]. Such an approach enabled the simultaneous grafting of PDMAEMA copolymer onto cellulose and crosslinking of the formed PDMAEMA by adding divinyl monomer. In the case of neat cellulose, the grafting of PDMAEMA is mainly performed onto the surface of microcrystalline cellulose particles or nanocellulose, which presents a significant limitation regarding the homogeneity of the system.

A novel approach that has been introduced in the last decades is radiation-induced graft polymerization, which has many advantages compared to classical chemical methods. The high penetrating ability of the photons makes the process much simpler, more accurate, and homogeneous; the process is temperature-independent, whereby local heating is avoided; and the reaction is easy to control, as only the applied dose and dose rate needs to be controlled. What is especially important regarding biomedical applications is that the process is clean and, if the reaction mixture is completely sealed, the final product is sterile when the applied dose is greater than 25 kGy.

Ionizing radiation was successfully used for grafting vinyl polymers (e.g., PDMAEMA, poly(acrylic acid), and polyvinylpirrolidone) onto the surface of various kinds of polymer materials such as poly(ethylene therephtalate), poly(vinylidene fluoride), cellulose, polypropylene, etc. [15,16,17,18,19,20,21]. Here again, it is important to note that in the case of cellulose, it was mostly fibers, micro-, or nanocellulose, i.e., grafting vinyl polymers onto neat cellulose is predominantly carried out under heterogeneous conditions with a water-based dispersant [22,23,24]. The only modification of water-soluble cellulose derivatives is carried out under homogeneous conditions [25,26]. To the best of our knowledge, there is no study on grafting vinyl polymers onto dissolved cellulose in non-aqueous media, such as DMAc/LiCl.

The structure of the crosslinked polymer network has a significant effect on the swelling behavior of hydrogels. Therefore, the study of the swelling behavior provides a deeper insight into the structure of the hydrogel. Furthermore, hydrogel swelling behavior is important in applications such as drug delivery, wound care, wearable electronics, etc. [27,28]. The preparation of neat cellulose and cellulose-based hydrogels with the desired swelling behavior is achieved by a tailored gelation or crosslinking methodology and modification with synthetic polymers [27,28,29,30]. The swelling degree of crosslinked materials is closely related to the mechanical and viscoelastic behavior of the materials. In hydrogels built from crosslinked linear polymers, the shear modulus depends on crosslinking density, i.e., the swelling degree. In contrast, if a hydrogel network is formed with grafted polymer molecules, the shear modulus depends not only on the crosslink density but also on the side chain length [31,32].

Additional functionality of hydrogels can be achieved by specific chemical reactions of hydrogels with metal ions or by adsorption of metal ions. It is known from the literature that the incorporation of silver (nano) particles contributes to the antibacterial properties of the material, which is especially important when hydrogels are used for biomedical purposes [33]. In addition, the specific adsorption of iron ions, which are widely distributed in the natural environment and biological system, can be beneficial for the development of hydrogels for wound treatment or sensors. Moreover, Fe^3+^ ions are involved in many human metabolic processes; for example, wound healing, in which the concentration of Fe^3+^ ions is an important parameter affecting the wound healing process. Additionally, the concentration of Fe^3+^ ions in the wound area is crucial for the prevention of bacterial growth in the wound area [34].

The present work aims to investigate the in situ incorporation of PDMAEMA/MBA onto cellulose backbone by free radical polymerization while preparing hydrogels of cellulose and PDMAEMA (cel-DMAEMA). The impact of DMAEMA content in the initial reaction mixture and subsequent use of ionizing radiation on reaction yield and grafting/crosslinking was investigated. Furthermore, the objectives of this study were to investigate the impact of hydrogel composition and gelation procedure on the swelling and viscoelastic properties, as well as on the morphology of the cel-DMAEMA hydrogels. Finally, in order to incorporate additional functionality, hydrogels were used as green reducing agents to introduce silver particles by a reduction in AgNO_3_. Additionally, the adsorption of Fe^3+^ ions was tested, aiming at future application, as wound dressing materials that inhibit bacterial growth by Fe^3+^ deprivation in the wound area. 

## 2. Results and Discussion

### 2.1. Polymerization Reaction

The copolymerization of *N,N-*dimethylaminoethyl methacrylate (DMAEMA) and *N*,*N*-methylenebis(acrylamide) (MBA) in cellulose solution is a complex process that can result in a number of different compounds [12,35,36,37,38], including (a) grafting a polymer onto cellulose, (b) forming a semi-interpenetrating network through crosslinking, (c) homopolymer or copolymer with a branched structure, and (d) polymer microgel. In this work, polymerization of DMAEMA and MBA was carried out in cellulose solution with different molar ratios of cellulose to DMAEMA (1:1, 1:3, and 1:5). The abbreviation of the prepared samples is 1-A B, where A denotes the molar ratio of cellulose and DMAEMA (1, 3, and 5) and B denotes the chemical polymerization (CP) and the additional radiation dose (10, 30, and 100). The total content of polymer in reaction mixtures was determined gravimetrically to investigate both the influence of the molar ratio of cellulose to DMAEMA and irradiation dose on the conversion of the polymerization reaction and the grafting/crosslinking of polymers. Furthermore, the opaque appearance of the reaction mixtures of poly(dimethylaminoethyl methacrylate) (PDMAEMA) polymerized in cellulose solution indicated the presence of a two-phase system in the mixtures after polymerization (Figures S1 and S2). Most likely, turbidity is a consequence of formed microgels. 

The presence of microgel particles in the reaction mixture was determined by optical microscopy, and obtained micrographs are shown in Supplementary Material Figures S3–S5. Microgel particles appeared after irradiation in 1-1 reaction mixtures, while in the case of 1-3 and 1-5 mixtures, microgel was observed in irradiated and non-irradiated samples. The size of microgel particles in 1-1 mixtures was up to 25 µm, while 1-3 and 1-5 mixtures showed microgel particles with a size of about 30 µm. Moreover, the microscopic images of 1-3 and 1-5 mixtures indicate that the concentration of particles in these reaction mixtures is much higher than in 1-1 mixtures. To separate and analyze the formed microgel particles, the reaction mixtures were diluted with *N*,*N*-dimethylacetamide (DMAc)/LiCl and centrifuged. An opaque precipitate of microgel particles (samples abbreviated P) was separated from a clear supernatant solution by centrifugation in the case of the 1-3 and 1-5 reaction mixtures. However, in the case of the 1-1 reaction mixtures, no precipitate was observed after centrifugation because the concentration of the microgel phase was too low and, therefore, difficult to separate quantitatively. The microgel particles were successfully separated in reaction mixtures 1-3 and 1-5, and their concentrations are presented in Supplementary Material Table S1. The lowest content of microgel particles was present in reaction mixture 1-3 CP, while increasing the DMAEMA content in the initial reaction mixture further promoted the formation of microgel particles (11.8 wt% in reaction mixture 1-5 CP). In addition, ionizing irradiation also promoted the formation of microgel particles. 

**Figure 1:**
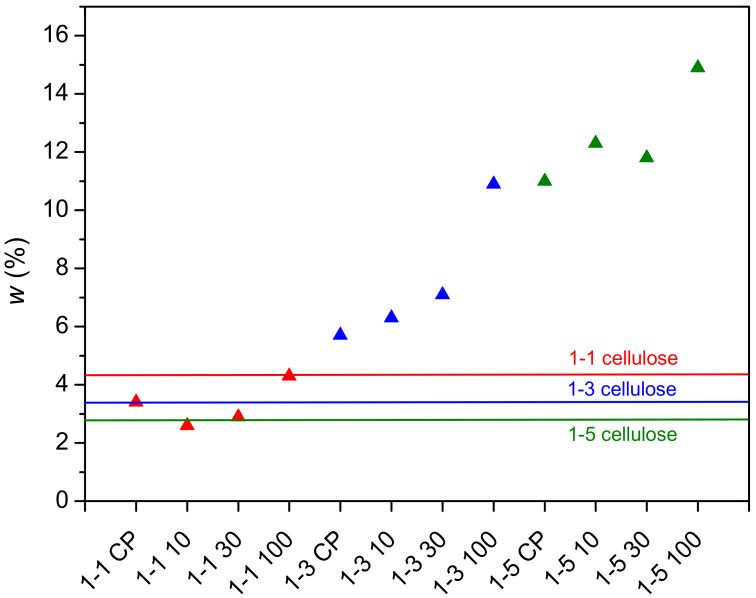
Concentration of the polymer in samples after polymerization of DMAEMA and MBA in cellulose solution (solid line—initial concentration of cellulose).

The total content of the polymer in mixtures after polymerization is shown in Figure 1. The initial concentration of cellulose in the reaction mixture for samples 1-1, 1-3, and 1-5 was 4.3 wt%, 3.4 wt%, and 2.8 wt%, respectively (solid lines in Figure 1). In studies in which PDMAEMA was grafted onto cellulose under heterogeneous conditions, the increase in polymer content is expressed as grafting efficiency [39,40]. Here, however, the increase/decrease in polymer content was expressed as polymer concentration in the reaction mixture due to the presence of microgel particles, which were not observed in heterogeneous conditions. Interestingly, polymer content for samples 1-1 (except 1-1 100) is lower than the initial concentration of cellulose in the reaction mixture, indicating that the degradation of cellulose occurred during the free radical polymerization. The random chain scission of cellulose molecules is most likely caused by degradation products originating from solvent DMAc/LiCl. Keteniminium salts, degradation products of DMAc, are formed at a temperature above 80 °C and, consequently, cause the degradation of cellulose [41,42]. In samples with a higher concentration of DMAEMA (molar ratio of cellulose to DMAEMA 1-3 and 1-5), the content of the polymer is higher than the initial content of cellulose, suggesting that the polymerization reaction prevails over the reaction of cellulose degradation. Moreover, the content of the polymer increased after gamma irradiation. The highest amount of the polymer was obtained at a radiation dose of 100 kGy in all samples (Figure 1).

### 2.2. Determination of PDMAEMA Content in Cel-DMAEMA Hydrogels

**Figure 2:**
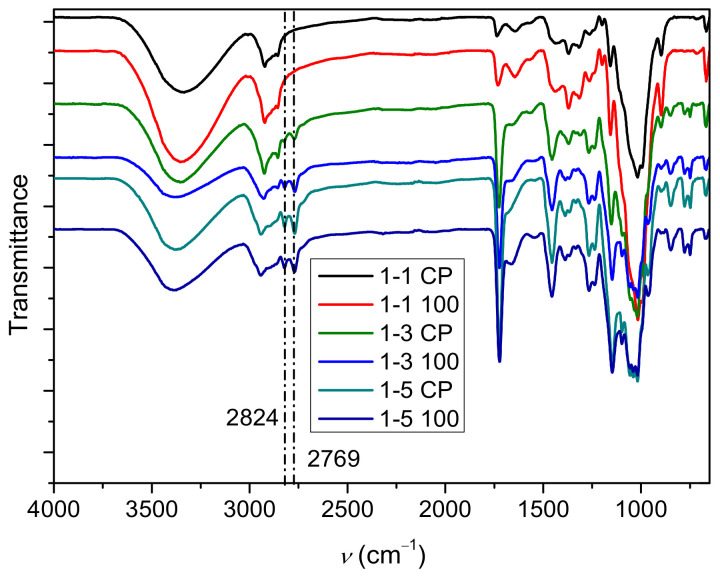
Infrared spectra of hydrogels prepared by polymerization and additionally irradiated samples.

**Figure 3:**
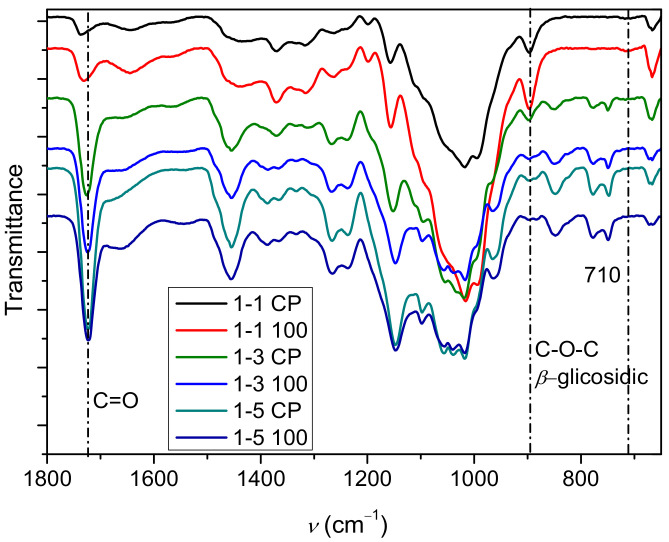
Infrared spectra of hydrogels prepared by polymerization and additionally irradiated samples (magnified).

The infrared spectra of the hydrogels 1-1 (CP, 100), 1-3 (CP, 100), and 1-5 (CP, 100) are shown in Figure 2 and Figure 3, while spectra of samples irradiated with lower doses are shown in Supplementary Material Figure S6. The relative ratio of cellulose to PDMAEMA in the hydrogel was determined by infrared spectra, as the content of the polymer in the prepared hydrogels changed with the change in the molar ratio of cellulose to DMAEMA and the irradiation dose.

The presence of PDMAEMA was revealed by the band at around 1724 cm^−1^, which can be assigned to carbonyl group vibrations [40,43]. Several other bands indicate the presence of PDMAEMA in the prepared samples, such as the vibrational bands at 2824 and 2769 cm^−1^ [43,44,45], which are characteristic of the CH_3_ group attached to the tertiary amine. These vibration bands are more pronounced in samples 1-3 and 1-5, indicating that the content of PDMAEMA increased relative to samples 1-1. In addition, a characteristic band of CH_2_ vibrations at 1457 cm^−1^ with a strong intensity also indicates the presence of PDMAEMA [45]. In the lower part of the spectrum, the bands at 780 and 750 cm^−1^ [45], which can be attributed to PDMAEMA, are more pronounced in the samples that had a higher content of DMAEMA in the initial reaction mixture (1-3 and 1-5 samples). All these bands indicate that there is a correlation between the content of PDMAEMA and the intensity of the bands.

The presence of MBA in the sample can be seen from the bands at 1644–1670 cm^−1^, which can be assigned to the vibrations of amide I, and from the bands at 1540–1560 cm^−1^, which can be assigned to the vibrations of the amide II bond [46,47,48]. The shift of the amide I band is strongly influenced by hydrogen bonds [49]. Other bands that can be assigned to the MBA overlap with other components present in the cel-DMAEMA hydrogel.

In addition, bands that can be assigned to functional groups present in the cellulose are also present in the IR spectra. A wide band at around 3400 cm^−1^ can be assigned to the OH groups of the cellulose and bound water molecules [50,51]. The vibrational bands of the C-O groups in cellulose are at about 1018, 1038, and 1056 cm^−1^ [52]. In sample 1-1 CP (highest cellulose content) the band at 1018 cm^−1^ has the highest intensity, while in sample 1-5 100 (lowest cellulose content), all three bands (1018, 1038, and 1056 cm^−1^) have similar intensity. Since the grafting of PDMAEMA during the polymerization reaction is to be expected, it can be assumed that the intensity of these bands changes due to the grafting of PDMAEMA molecules onto the cellulose chain. Furthermore, a change in the intensity of the β-glycosidic bonds in the cellulose (897 cm^−1^) was also observed with the change in cellulose content in the cel-DMAEMA samples.

**Figure 4:**
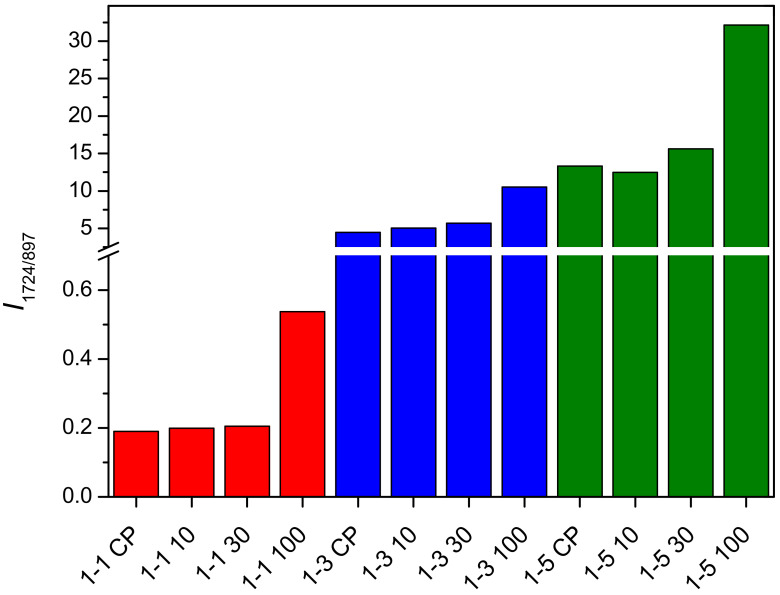
Intensity ratio of PDMAEMA band (1724 cm^−1^) and cellulose band (897 cm^−1^) for prepared samples.

The bands assigned to vibrations of the carbonyl group in PDMAEMA (1724 cm^−1^) and the β-glycosidic bonds in cellulose (897 cm^−1^) were selected for the evaluation of the relative composition of cel-DMAEMA hydrogels because these two bands do not overlap with the bands of other functional groups present and have a relatively high intensity. The intensity ratios for samples 1-1, 1-3, and 1-5 are shown in Figure 4. The intensity ratio increases as the feeding amount of DMAEMA increases, indicating a higher content of PDMAEMA in the material. Moreover, a significant increase in the intensity ratio (135–185%) was observed at the largest applied dose (100 kGy) of gamma irradiation relative to non-irradiated samples (CP). Although EDS analysis is used for the determination and mapping of heavy atoms, for the prepared cel-DMAEMA samples, it was possible to determine the nitrogen content by semi-quantitative EDS analysis (Table 1). The results obtained for the nitrogen content in the cel-DMAEMA samples were used to further support the relative content of the cel-DMAEMA samples obtained from the FTIR measurements. In sample 1-1 CP nitrogen was not determined, probably due to the content being lower than the EDS detection limit while other samples displayed an increasing trend compared to the intensity ratio of FTIR bands. 

**Figure 5:**
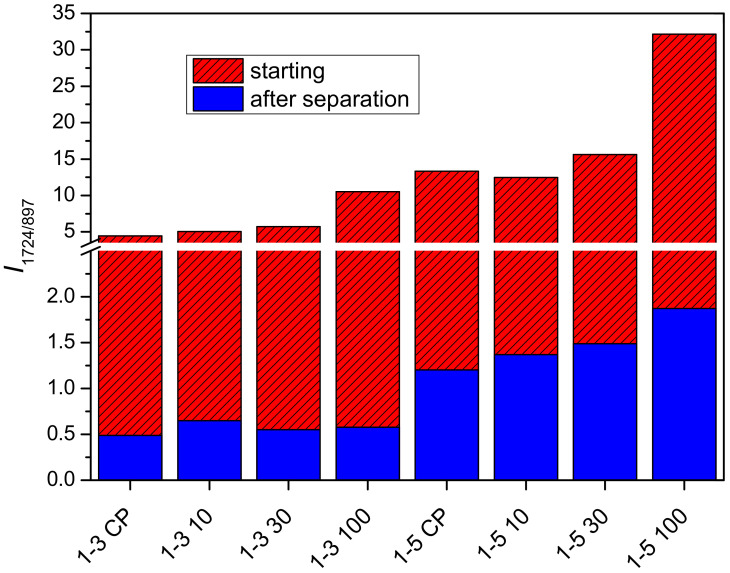
Intensity ratios of PDMAEMA band (1724 cm^−1^) and cellulose band (897 cm^−1^) for polymers obtained from supernatant solution (after separation—samples with abbreviation F).

The purified and dried polymer of the microgel particles (abbreviation P) and the polymer obtained from the supernatant solution (abbreviation F) of samples 1-3 and 1-5 were also characterized by FTIR. Samples 1-1 were omitted because the microgel particle content was too low to be quantitatively separated. Characteristic bands for PDMAEMA (Supplementary Material Figures S7–S10) (2824, 2769, and 1724 cm^−1^) were observed in the polymer of the microgel particles, while bands characteristic for cellulose (897 and 1000–1150 cm^−1^) were not observed. The band around 3300 cm^−1^ can be assigned to water present in the precipitate sample due to the hydrophilic nature of PDMAEMA. These results indicate that the obtained microgel is formed from PDMAEMA. In a similar study in which PDMAEMA was grafted onto cellulose under homogeneous conditions (concentrated phosphoric acid), opaque hydrogels were obtained. However, a detailed study of the composition of the phase causing the opaque appearance has not been carried out [53]. In the FTIR spectra of the polymer obtained from the supernatant solution (abbreviation F), the characteristic bands of PDMAEMA and cellulose are present (Supplementary Material Figures S11–S14). The intensity ratio of characteristic bands of PDMAEMA (1724 cm^−1^) and cellulose (897 cm^−1^) in 1-3 F and 1-5 F samples are shown in Figure 5. Comparing the FTIR bands of the initial samples (1-3 and 1-5) with the 1-3 F and 1-5 F samples, the latter display a 10–18 times lower intensity ratio. Such a decrease in the intensity ratio for samples 1-3 F and 1-5 F is expected given that a large amount of crosslinked PDMAEMA in form of a separated microgel was removed (samples with the abbreviation P). After gamma irradiation, the intensity ratio for samples 1-3 F and 1-5 F increased by 10–34% and 14–45%, respectively (Figure 5). This increase in intensity ratio indicates that gamma irradiation promotes the grafting of PDMAEMA onto cellulose.

### 2.3. Influence of Composition and Irradiation on Swelling Behavior of Hydrogels

**Figure 6:**
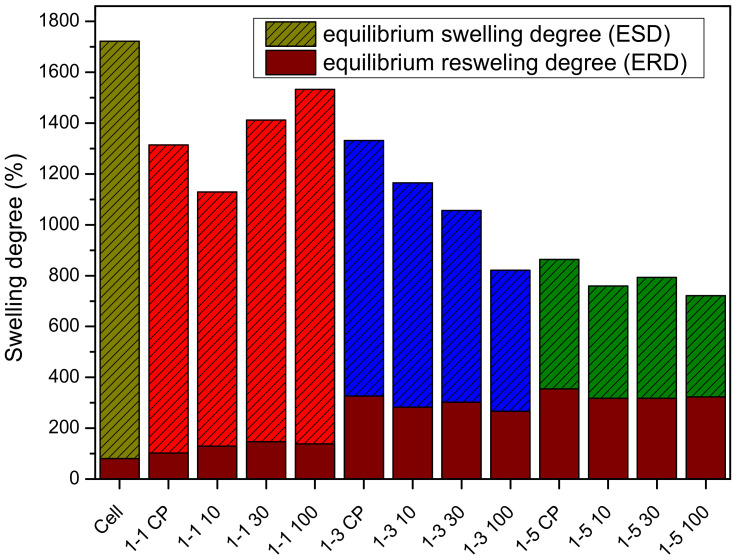
Swelling degree for neat cellulose and prepared hydrogel samples.

The swelling behavior of hydrogels prepared from reaction mixtures was studied to gain insight into the contribution of material composition to the degree of swelling/reswelling. Hydrogels of neat cellulose, 1-1, 1-3, and 1-5 samples were prepared in the form of spheres and the equilibrium swelling degree (ESD) was determined (Figure 6).

A lower equilibrium swelling degree is observed for all CP samples relative to neat cellulose. The ESD of CP samples was only affected at the highest molar ratio of cellulose to DAMEMA (1:5). Moreover, the ESD was affected by the dose of gamma irradiation. The ESD increased with the dose for samples 1-1, except for samples 1-1 10, while the irradiation dose had the opposite effect for samples 1-3 and 1-5. It is worth noting that only a negligible amount of PDMAEMA microgel was observed in samples 1-1, indicating that formed PDMAEMA is grafted onto cellulose. Thus, the increase in grafting yield with the increase in dose could be a possible reason for the increase in the ESD in 1-1 samples. In the case of 1-3 and 1-5 samples, the irradiation dose may affect the grafting yield and crosslinking density of the PDMAEMA microgel (samples with the abbreviation P). The overall change in the ESD then depends on the ratio of the relative increase in grafting yield and the relative increase in crosslinking density of the PDMAEMA microgel. In general, higher crosslinking density decreases the swelling degree of hydrogels [54]. It can be assumed that the relative increase in crosslinking density of PDMAEMA microgel particles in irradiated samples 1-3 and 1-5 could be the reason for the decrease in the ESD.

Neat cellulose and synthesized samples were reswollen after drying to determine the equilibrium reswelling degree (ERD) because cellulosic materials exhibit low reswelling after drying due to intra- and intermolecular hydrogen bonding [52]. The grafting of polymer molecules onto cellulose would reduce the intra- and intermolecular hydrogen bonds and thus improve the reswelling of the material. The degree of reswelling of pure cellulose and all samples was much lower than the initial ESD of each sample (Figure 6). Cellulose had the lowest reswelling degree (4.6% of the initial ESD). ERD for samples 1-1 was 8–10% of the initial ESD, while ERD for samples 1-3 and 1-5 was 24.5–32.5% and 40.0–44.8% of the initial ESD, respectively. The higher ERD is the result of the higher amount of PDMAEMA in samples 1-3 and 1-5, which is evident from the FTIR data before and after the removal of the PDMAEMA microgel.

**Figure 7:**
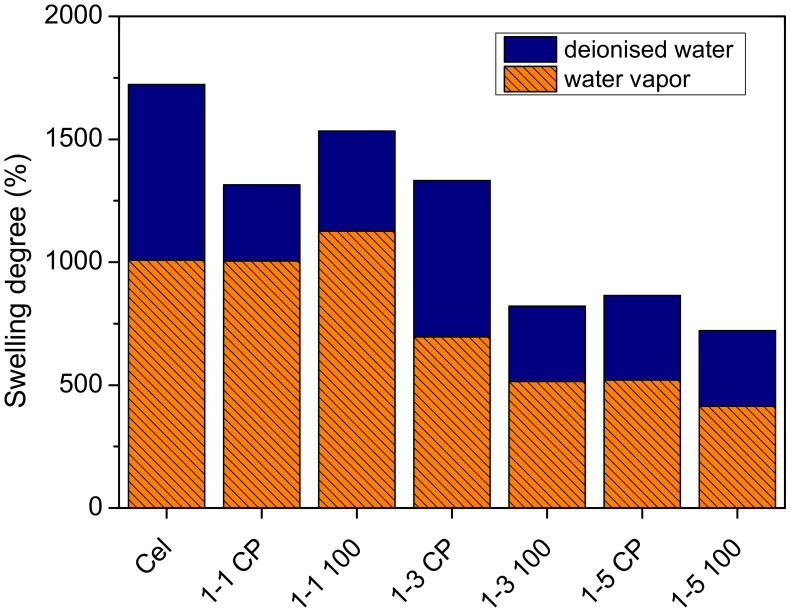
Swelling degree of hydrogels prepared with direct immersion in deionized water and hydrogels gelled with water vapor.

Furthermore, gelation of neat cellulose solution and cel-DMAEMA solutions (both samples with abbreviation CP and 100) was carried out with water vapor and compared with direct gelation of solutions in the deionized water in order to investigate the influence of the gelation method on the swelling degree of prepared gel since the swelling degree can affect viscoelastic and mechanical properties [32]. By exposing the cellulose solution to the water vapor, the thermodynamic quality of the solvent gradually deteriorates with the gradual mixing of water in DMAc/LiCl, thus affecting the molecular conformation and gelling process. On the other hand, when the cellulose solution is directly immersed in deionized water, the thermodynamic quality of the solvent is rapidly changed by the fast mixing of water in DMAc/LiCl. The equilibrium swelling degree of neat cellulose hydrogel was significantly lowered by gelling in water vapor (ESD = 1009%) in comparison to direct gelation in deionized water (ESD = 1720%) (Figure 7). The same effect was observed in the case of cel-DMAEMA solutions regardless of PDMAEMA content (Figure 7). Therefore, it is evident that the rate of solvent exchange plays a crucial role in the control of the swelling degree of cellulose hydrogels prepared by physical crosslinking. Although the swelling degree was significantly decreased by the water vapor method of gelation, this method has proven to be effective for the preparation of hydrogels in the form of films while the direct immersion method was more suitable for the preparation of hydrogels in the form of spheres. 

### 2.4. Rheological Properties of Prepared Hydrogels

**Figure 8:**
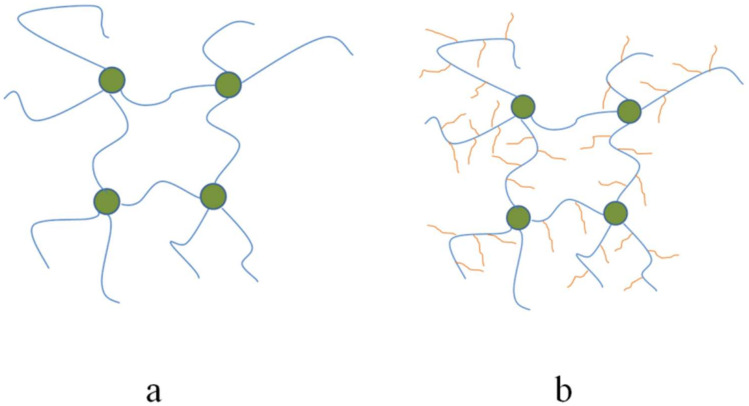
Schematic illustration of an ideal polymer network with linear chain strands (**a**) and a polymer network with grafted chain strands (**b**).

The dynamic viscoelastic behavior of cellulose and cel-DMAEMA hydrogels prepared in the form of films was studied in order to correlate the composition of the prepared hydrogel to its viscoelastic properties. Physical gels, such as the cellulose gels prepared in this work, exhibit transient crosslinks formed by interchain associations involving OH groups that exhibit hydrogen bonding. Moreover, the crosslinks in physical cellulose gels are created through formed crystallized segments [31,55,56]. The classical rubber elasticity theory was initially developed to understand solvent-free rubber materials; however, rubber elasticity theory is often applied to understand the mechanical properties of hydrogels. The structure of polysaccharide-based hydrogels (cellulose, alginate, and chitosan), as well as protein-based hydrogels, is often correlated to their mechanical properties. The rubber elasticity theory correlates the shear modulus, one of the important physical properties of polymer gels, with the concentration (number) of elastically active chains in the polymer network [57]. In addition, the modulus of elastomeric and gel materials is affected by the presence of dangles and loops, which are typical by-products of the crosslinking process. In the case of polymer networks formed by graft polymers (Figure 8) the degree of polymerization of the side chain, the graft density, and different topologies of side chains have a significant effect on the modulus of the formed elastomer or gel [31,32].

**Figure 9:**
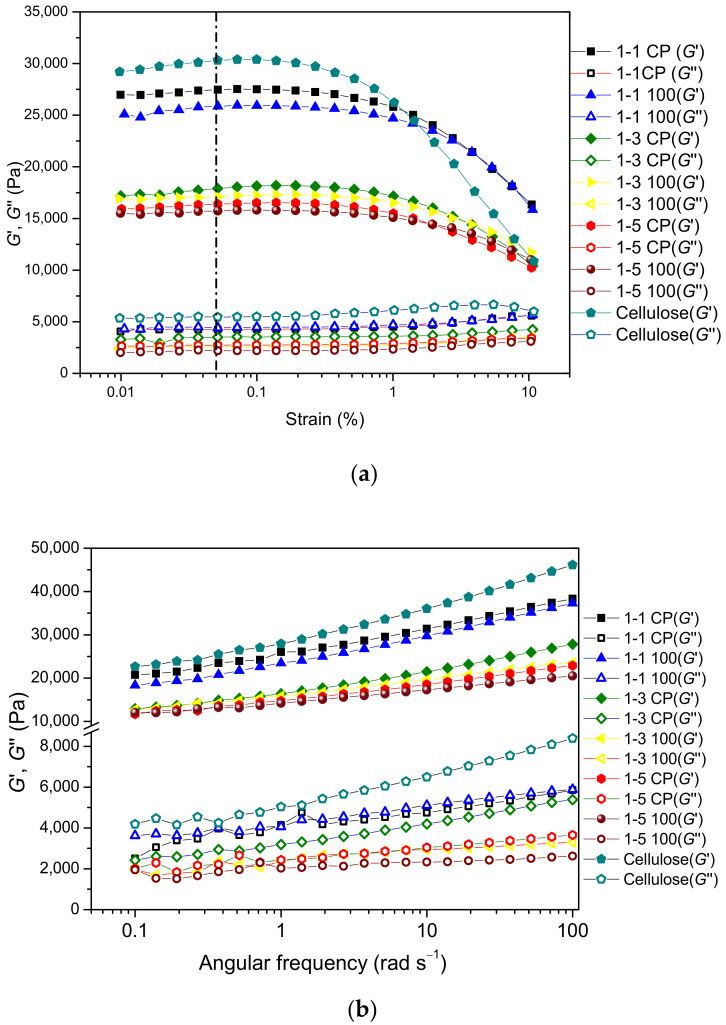
Storage (*G*′) and loss modulus (*G*″) of prepared hydrogels in an amplitude sweep test (**a**) and a frequency sweep test (**b**).

To determine the linear viscoelastic region (LVER), the strain sweep test was performed at a fixed frequency (the region in which the modules are strain-independent) to determine the critical strain for the analyzed hydrogels. Figure 9a shows the results of the amplitude sweep test. The storage modulus of neat cellulose and cel-DMAEMA hydrogels in LVER is in the range of 15–30 kPa, which is in the range of chemically crosslinked cellulose-based hydrogels [58,59]. The critical strain was determined from the graph of the storage modulus *G*′ versus strain amplitude (Figure 9a), and the values are listed in Supplementary Material Table S3. Beyond the value of critical strain (*γ*_c_), the viscoelastic module decreased as a consequence of the structural breakdown of the hydrogel network under high deformation [60]. Modification of cellulose hydrogel with PDMAEMA contributes to the increase in critical strain (*γ*_c_). In addition, ionizing irradiation, which increased the amount of PDMAEMA in the hydrogel, further improved the critical strain of prepared hydrogels. 

**Figure 10:**
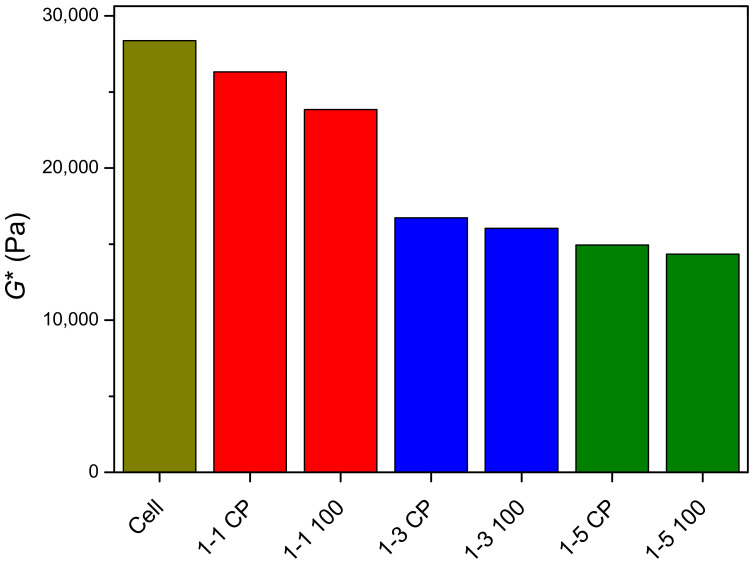
Complex modulus (G*) of prepared hydrogels determined at a frequency of 1 rad s^−1^.

In Figure 9b, frequency sweep tests are summarized for analyzed hydrogels. Throughout the frequency range, neat cellulose and cel-DMAEMA hydrogels exhibit larger storage modulus (*G*′) than loss modulus (*G*″), indicating gel-like viscoelastic behavior. Storage and loss modulus of all hydrogels show a weak linear dependence on frequency, which is characteristic of a gel-like material [61]. For a better comparison of hydrogels’ viscoelastic properties, the complex modulus (G*) was determined at a frequency of 1 rad s^−1^ and the results are shown in Figure 10. Neat cellulose hydrogel has the highest G* value, indicating the highest stiffness among the analyzed hydrogels. The introduction of PDMAEMA in cellulose-based hydrogel adds to the complexity of the correlation of the polymer network structure and physical properties since both the shear modulus and swelling degree are strongly influenced by the grafting density and length of side chain [62]. Furthermore, the presence of PDMAEMA modifies the formation of the hydrogel network and the crystallization of cellulose. The influence of the polymer network structure was revealed from rheological characterization since the stiffness of 1-1 CP hydrogel decreased (7.2%) relative to neat cellulose hydrogel. In contrast, the ESD of these hydrogels did not show any influence of the polymer structure since the values of the ESD of neat cellulose and 1-1 CP hydrogel were close. The molecular weight between crosslinks of 1-1 CP hydrogel did not significantly change relative to *M*_c_ of cellulose hydrogel, indicating that the grafting of a small amount of PDMAEMA did not significantly affect crosslink density in 1-1 CP hydrogel.

In systems such as these studied here, ionizing irradiation can promote crosslinking of polymer chains, chain scission, and grafting. Crosslinking in hydrogels with linear polymer strands induced by ionizing radiation is evident from a decrease in swelling degree and an increase in the shear modulus [63]. On the contrary, in polysaccharide hydrogels prepared by ionizing radiation chain scission is evident from an increase in molecular weight between crosslinks which is often accompanied by an increase in swelling degree and a decrease in the shear modulus [64]. Here, an increase in irradiation dose in 1-1 samples increased molecular weight between crosslinks (Table 2) compared to non-irradiated samples (1-1 CP), indicating lower crosslink density, while the ESD of prepared 1-1 100 films increased. Such behavior indicates that ionizing irradiation in the case of 1-1 100 also contributed to the chain session. 

The molar ratio of cellulose to DMAEMA monomer in the initial reaction mixture had a crucial role in the grafted PDMAEMA content and the PDMAEMA microgel content which further altered the mechanical properties of the prepared hydrogels. The presence of microgel particles in samples 1-3 and 1-5 significantly affected molecular weight between crosslinks since it was reduced 1.5–1.9 times compared to the neat cellulose hydrogel (Table 2). Furthermore, complex modulus (G*) for 1-3 and 1-5 samples decreased relative to neat cellulose hydrogel (41.0–49.5%) following such a decrease in *M*_c_. Ionizing radiation had a negligible effect on the *M*_c_ for samples 1-3 but increased the crosslink density for samples 1-5. However, further an increase in microgel content by the irradiation also affected the G* of the hydrogels by disrupting the crosslink density of cellulose and grafted PDMAEMA molecules.

**Figure 11:**
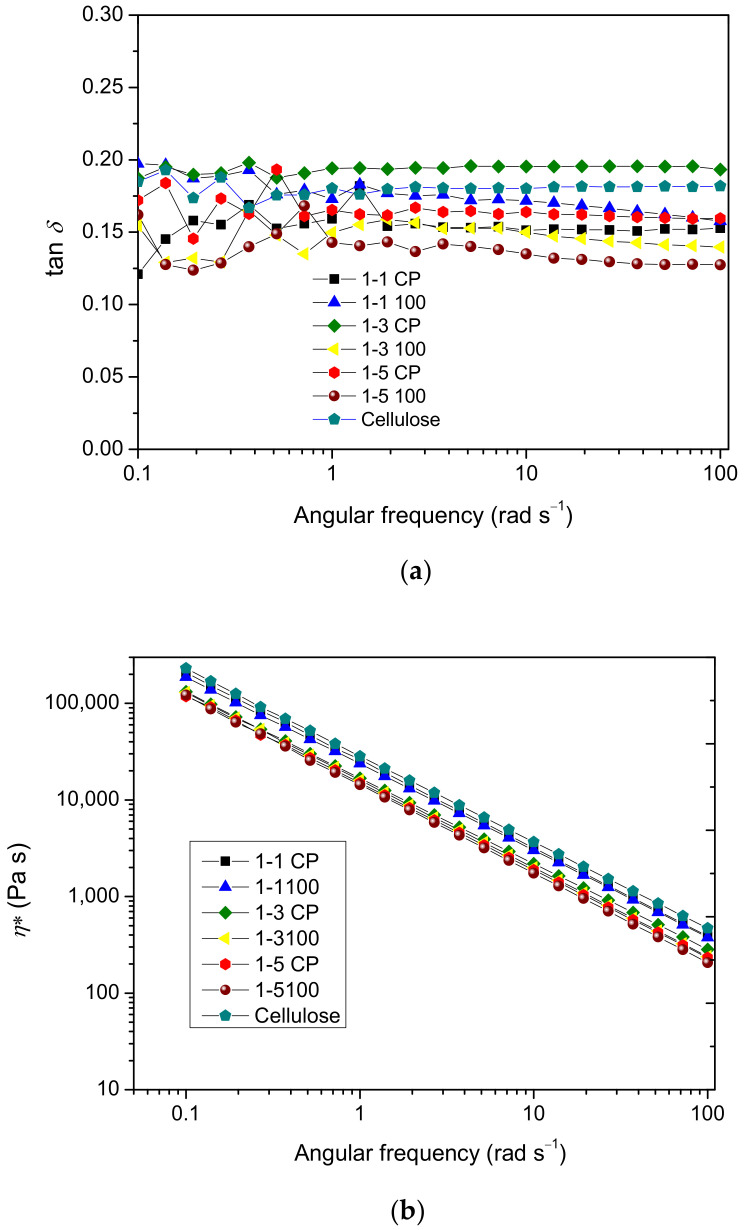
Frequency dependence of tan *δ* (**a**) and complex viscosity (η*) (**b**) of prepared hydrogels.

The values of tan *δ* (Figure 11a) are in the range of 0.12–0.20 and remain quite constant over the studied frequencies for all hydrogels. Such behavior is characteristic of solid-like materials. The decrease in tan *δ* values indicates the increasing elasticity since tan *δ* represents the ratio of dissipated and stored energy. Hydrogel 1-5 100 had the highest elasticity among all analyzed samples based on the lowest tan *δ* values. The absolute values of the slope of log η* versus the log *ω* of the prepared hydrogels (Figure 11b) ranged from 0.89–0.92, indicating solid-like behavior.

### 2.5. Morphology of Prepared Hydrogels

**Figure 12:**
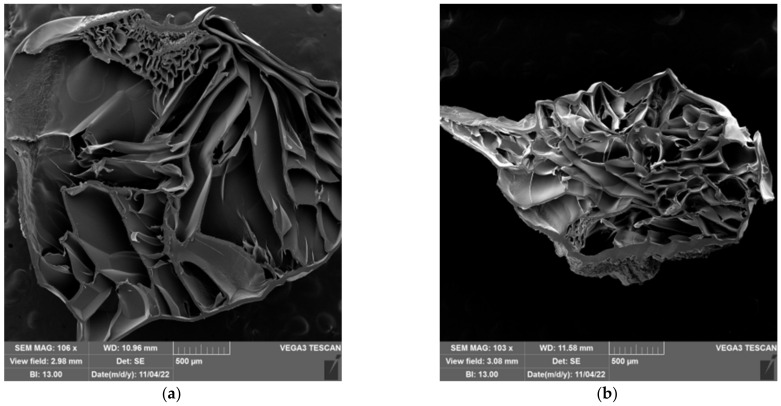

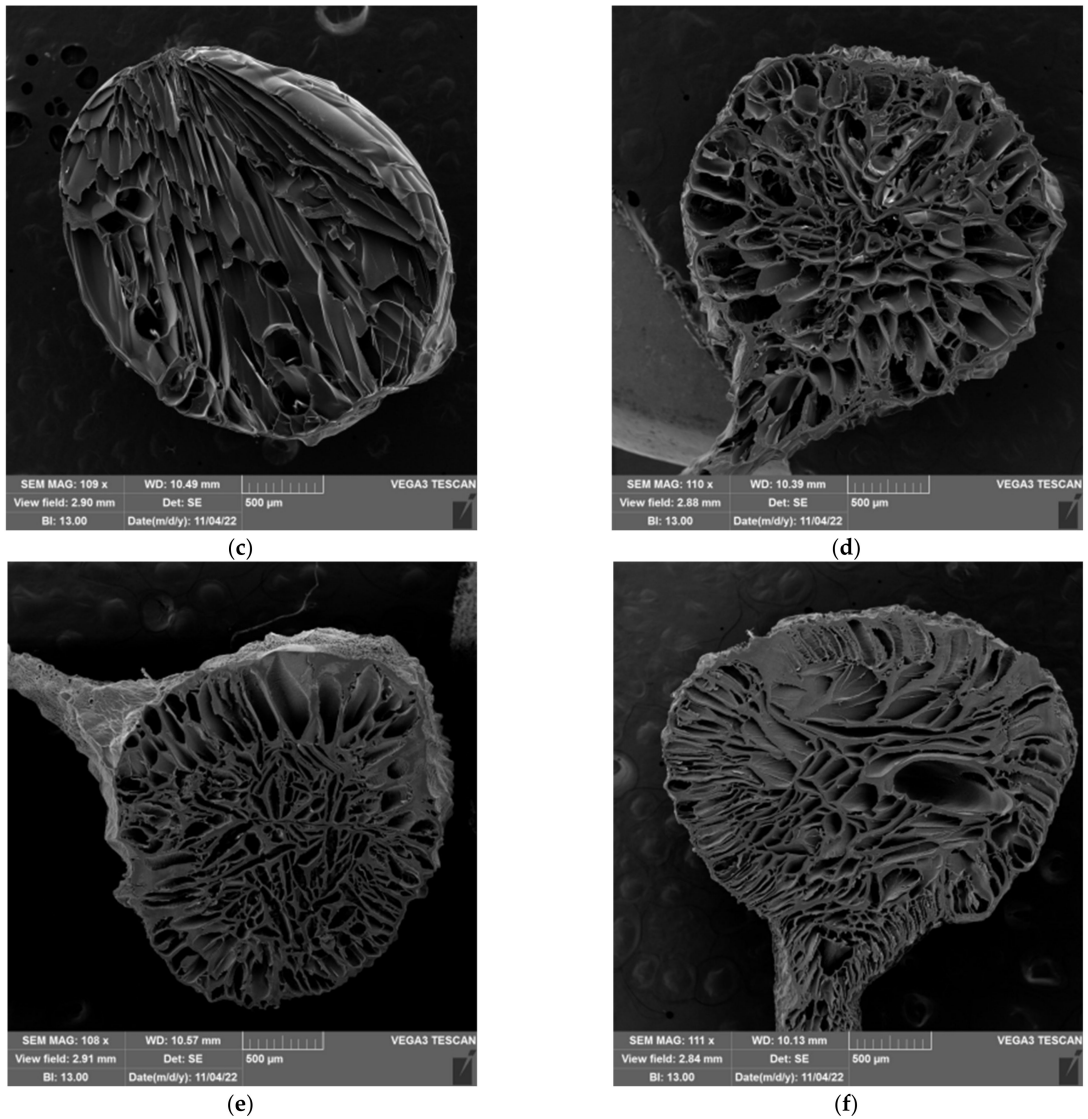

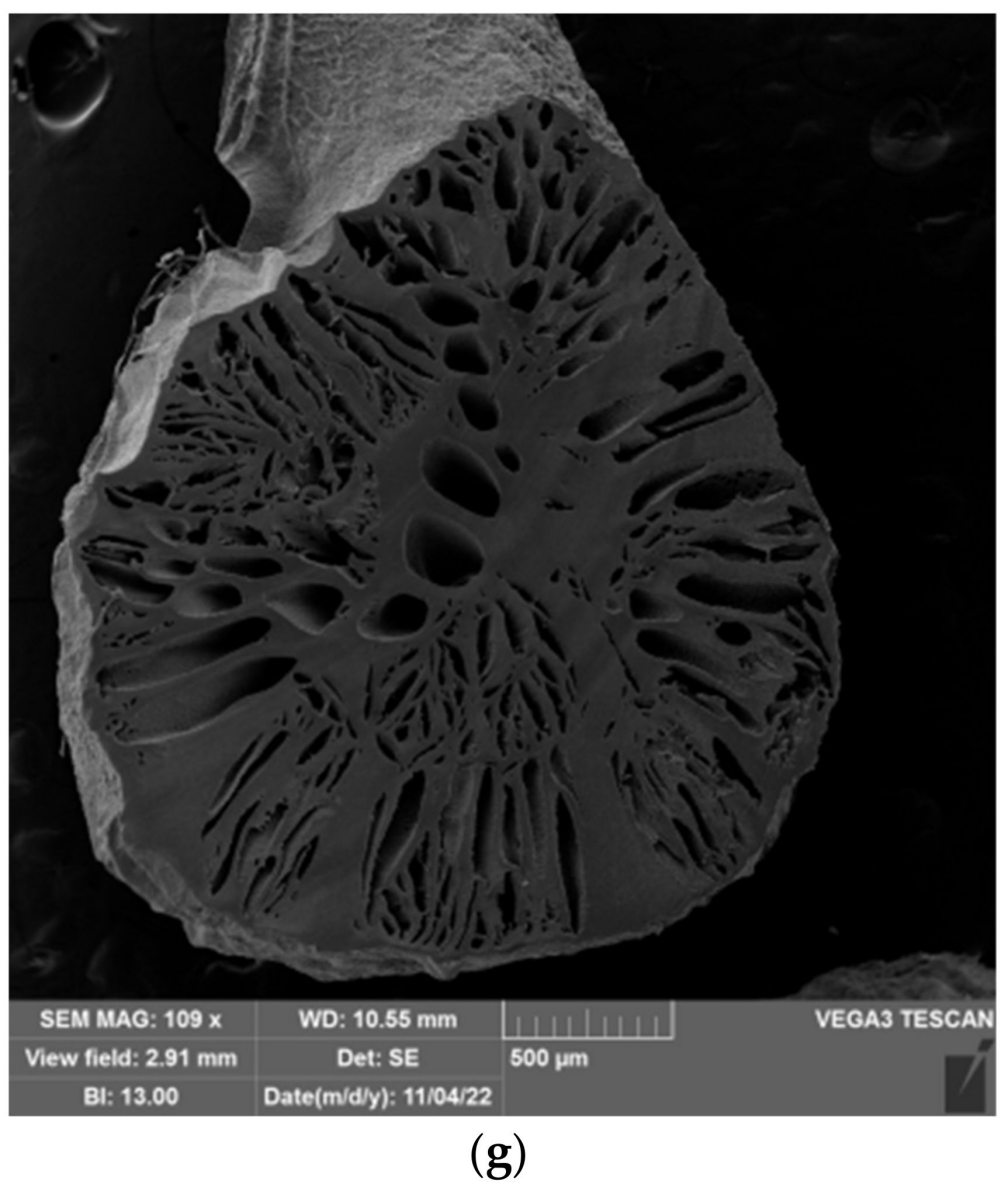
SEM micrographs of a cross-section of the sphere for cellulose samples (**a**), 1-1 CP (**b**), 1-1 100 (**c**), 1-3 CP (**d**), 1-3 100 (**e**), 1-5 CP (**f**), and 1-5 100 (**g**).

**Figure 13:**
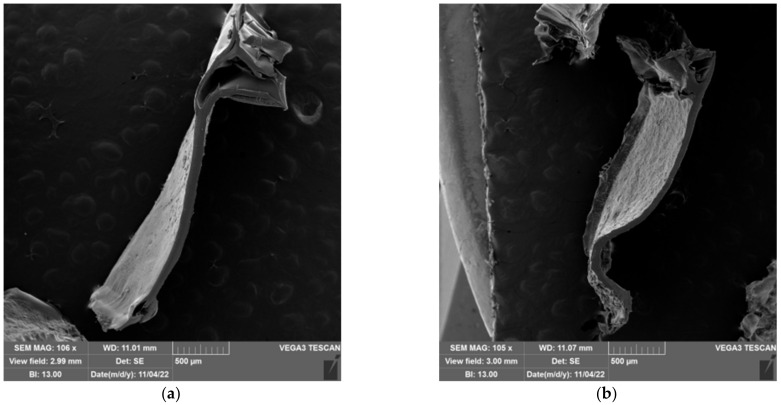

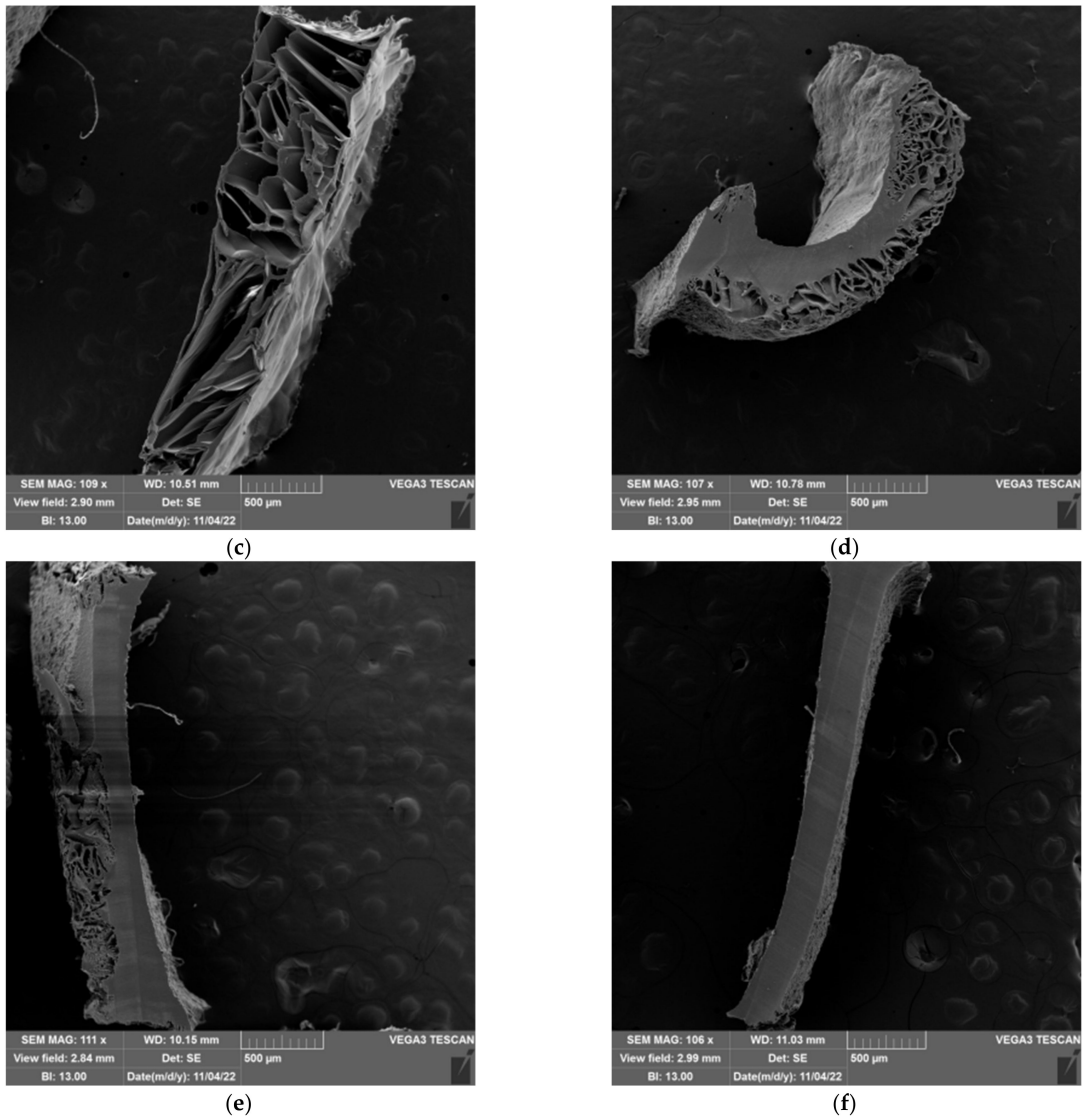

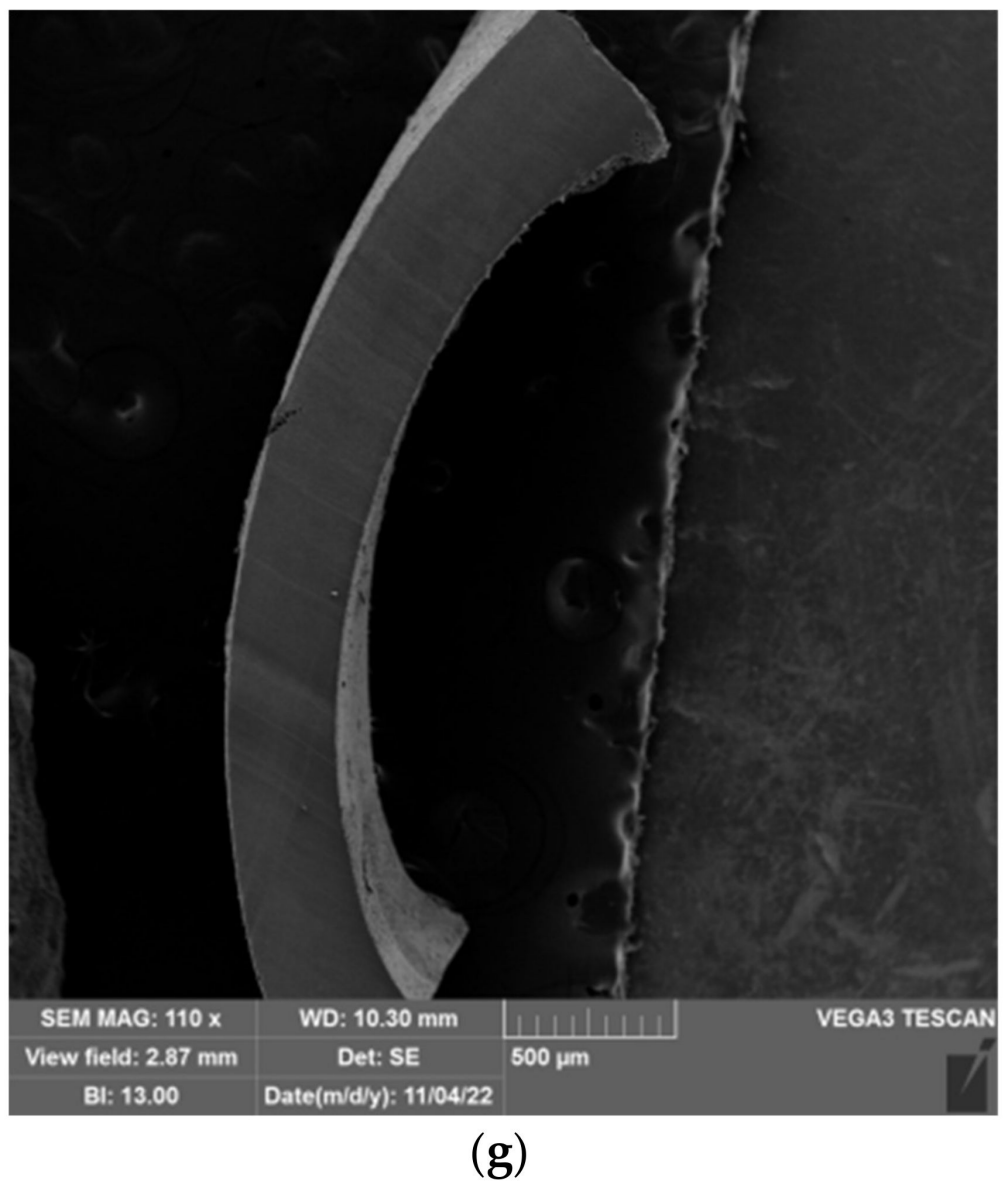
SEM micrographs of a cross-section of films for cellulose samples (**a**), 1-1 CP (**b**), 1-1 100 (**c**), 1-3 CP (**d**), 1-3 100 (**e**), 1-5 CP (**f**), and 1-5 100 (**g**).

The freeze-extraction method was successfully used to prepare porous structures from polysaccharides (chitosan and cellulose acetate) [65,66,67] and synthetic polymers (polyacrylonitrile and polylactic acid) [68,69]. These materials were later used as scaffolds for tissue engineering, as adsorbents, and as the active layer of separation membranes. In the aforementioned works, polymeric solutions were used to produce porous structures. Thermally induced polymer separation and solvent freezing were used to create porous structures [66,67,68,69]. Here, however, previously prepared hydrogels were used to shape porous material. The influence of the water content (expressed by the ESD) and the composition of the hydrogel on the pore structure were investigated. The morphology of the prepared spheres and films was characterized by scanning electron microscopy (SEM). SEM microscopic images of the prepared spheres and films are shown in Figure 12 and Figure 13. In the neat cellulose sphere, a porous structure was obtained with large elongated pores (150–400 µm) dominating the cross-section. In samples 1-1, large (70–300 µm) elongated pores were also present in the cross-section of the spheres. Increasing the PDMAEMA content affected the shape of the pores formed, i.e., pores of more uniform size were observed across the cross-section; although, shorter and elongated pores were still observed. In addition, pores with thicker pore walls were formed in the samples with the higher PDMAEMA content, which is particularly evident in samples 1-5 100 (Figure 12g).

The size distribution of the pores in the samples of cel-DMAEMA changes over the cross-section of the sphere. In the parts of the outer layer of the sphere, there are smaller pores (20–50 µm), while the pores in the center of the sphere are larger. The smaller pores are the result of the formation of smaller water crystals formed during the freezing process. 

Prepared films from cel-DMAEMA samples contained a lower amount of water compared to cel-DMAEMA spheres. The water content in the hydrogel influenced the formation of a porous structure, as a porous structure was not observed in all films. The neat cellulose film and 1-1 CP film contained the same amount of water in the hydrogel and in both samples dense films were obtained after freeze-extraction. However, the porous structure across the entire cross-section was observed in the film of 1-1 100 samples, which had higher water content. A porous structure was observed in a small area of the films of 1-3 samples, although the amount of water in 1-3 samples decreased by 30–50% compared to cellulose and 1-1 CP. Since the shear modulus was also significantly affected by the composition of the cel-DMAEMA films, it can be assumed that this also influenced the formation of the porous structure of the 1-3 samples. Further, an increase in PDMAEMA content and a decrease in water content in 1-5 CP and 1-5 100 did not result in a porous structure of the films, as shown in Figure 12f and Figure 12g, respectively.

### 2.6. Hydrogels with Silver Particles

**Figure 14:**
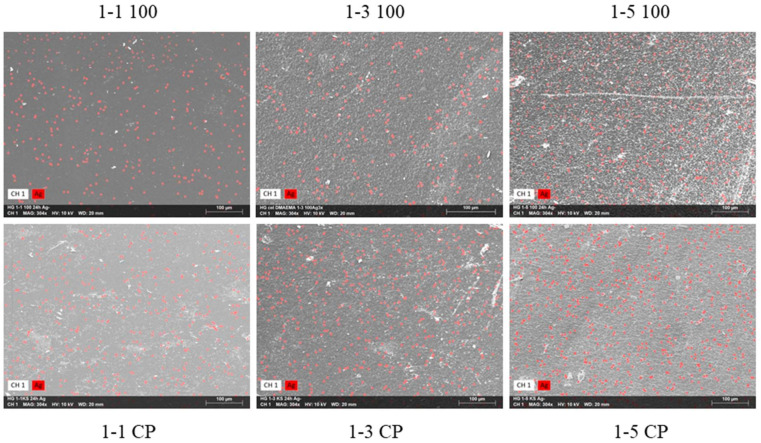
SEM micrographs with mapped Ag particles on the surface of the prepared hydrogel.

The incorporation of PDMAEMA into hydrogels may improve the antibacterial properties of the prepared material since PDMAEMA has shown good antibacterial properties in previous studies [33]. PDMAEMA has amino groups in the structure, which can be used for the synthesis of silver nanoparticles from silver ions without an additional chemical reducing agent [6,33]. In addition, a reduction in silver ions may also occur in hydroxyl groups in cellulose [70]. The control of the size and shape of the silver particles is enabled by the polymer network in the hydrogel, as the free spaces between the polymer molecules act as nanoreactors [71,72]. Furthermore, the polymer network stabilizes particles, thus avoiding the agglomeration of formed particles [72]. In our previous work, it was found that cel-DMAEMA hydrogels enable a reduction in Ag^+^ ions, thus forming Ag particles, which contributed to the antibacterial properties of hydrogels [73]. Here, the influence of PDMAEMA content in hydrogels on the distribution and content of formed Ag particles was investigated in more detail. First, the reaction of neat cellulose hydrogel with AgNO_3_ was carried out. After the reaction with silver nitrate, the hydrogel of neat cellulose turned gray, while the color of the hydrogels with PDMAEMA turned brown (Supplementary Material Figures S15–S17). Interestingly, for samples 1-3 and 1-5, the color intensity changed from a darker brown to a lighter brown when the irradiation dose increased. The content of Ag particles in the hydrogels was determined by the semi-quantitative method SEM-EDS. The results are shown in Table 3, while micrographs with mapped Ag particles are shown in Figure 14. Silver particles were observed in neat cellulose, as well as in samples containing PDMAEMA, while Ag particles were equally distributed across the surface of the samples (Figure 14). The incorporation of PDMAEMA into the hydrogel (samples 1-1 CP) slightly increased the content of Ag particles relative to neat cellulose (Table 3). However, samples with higher PDMAEMA content (1-3 CP and 1-5 CP) had higher Ag content relative to neat cellulose. As can be seen from the results, hydroxyl groups from cellulose enabled the synthesis of Ag particles; however, with the addition of amino groups (PDMAEMA), the content of formed Ag particles significantly increased (Table 3).

### 2.7. Adsorption of Fe^3+^ Ions

**Figure 15:**
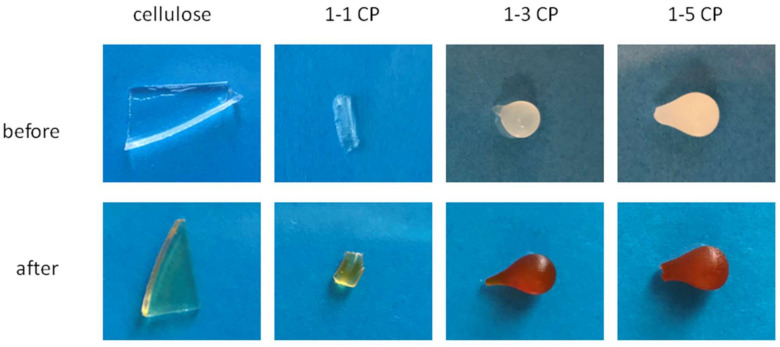
Prepared hydrogels before and after the adsorption of Fe^3+^ ions.

The presence of various functional groups, especially nitrogen, makes hydrogels promising adsorbents for the purification of water from heavy metals. Additionally, the possibility of specific adsorption of metal ions allows the development of hydrogels with sensory properties or advanced materials for wound treatment [34,74,75]. Moreover, the presence of Fe^3+^ ions in the wound area allows the growth of pathogens. The removal of metal ions, such as Fe^3+^, from the wound area represents a new approach to the development of antibacterial hydrogels for wound treatment [34]. Here, adsorption of Fe^3+^ was performed with hydrogels of neat cellulose, 1-1 CP, 1-3 CP, and 1-5 CP, and the results of adsorption are shown in Figure 15. The adsorption of Fe^3+^ ions was easily seen in samples 1-3 CP, and 1-5 CP by the strong color change (Figure 15). The cellulose samples and sample 1-1 CP did not show a strong color change, indicating a much lower adsorption capacity compared to samples 1-3 CP and 1-5 CP. These results indicate that PDMAEMA strengthens Fe^3+^ adsorption, as samples 1-3 CP and 1-5 CP have a higher amount of PDMAEMA according to FTIR analysis. The results obtained in these Fe^3+^ adsorption experiments are a good starting point for future detailed studies on the preparation of cel-DMAEMA hydrogels for use in wound treatment.

## 3. Conclusions

Polymerization of DMAEMA/MBA in cellulose solution revealed that the reaction of the vinyl/divinyl monomer system is a complex process with more than one product formed. FTIR analyses of the separated components formed in the reaction have shown that two processes occur during the polymerization of DMAEMA and MBA in cellulose solution: the grafting of PDMAEMA onto cellulose and the formation of PDMAEMA microgel particles. It was also observed that gamma irradiation promotes the grafting of PDMAEMA polymer onto cellulose, but complete crosslinking was not achieved in the dose range of 10–100 kGy. The structure and content of PDMAEMA affected the degree of swelling and reswelling of the prepared hydrogels. The increase in the grafting yield increased the equilibrium swelling degree of the 1-1, while in the samples of series 1-3 and 1-5, the presence of microgel particles caused a decrease in the ESD. The dynamic viscoelastic measurements reveal that the complex modulus of prepared hydrogels is in a range of 14–39 kPa, which is in a range for hydrogels suitable for biomedical use. Furthermore, the complex modulus and stiffness of cel-DMAEMA hydrogels were influenced by the content of PDMAEMA in the hydrogel. Finally, it was shown that the increasing PDMAEMA content has a positive effect on the synthesis of Ag particles in the hydrogel. Additionally, the increased Fe^3+^adorption capacity of hydrogels modified with PDMAMEMA opened an opportunity for the prospective development of hydrogels for wound treatment.

## 4. Materials and Methods

### 4.1. Materials

The stabilizing agent was removed by passing the monomer through a column containing activated basic alumina (Sigma-Aldrich, St. Louis, MO, USA) before using the monomer *N*,*N*-dimethylaminoethyl methacrylate (DMAEMA) (Sigma-Aldrich). In addition, the monomer was purged with nitrogen before use. The crosslinking agent *N*,*N*-methylenebis(acrylamide) (MBA, Sigma-Aldrich, purity 99%) was used without additional purification. The initiator of the polymerization reaction tert-butylperoxy-2-ethylhexanoate (Akzo Chemie, Trigonox 21, 70 wt% solution) was used without additional purification. The solvent *N*,*N*-dimethylacetamide (DMAc) (Fischer Chemical, analytical reagent grade) and lithium chloride were used to dissolve cellulose (*M*_v_ = 25,100 g mol^−1^) (Fischer Chemical, laboratory reagent grade). Absolute ethanol (Gram-mol, p.a. purity) was used for the freeze-extraction process without further purification. Cellulose was first vacuum dried over phosphorus pentoxide (VWR, 99.5% purity) for 3 h at 90 °C before dissolution in DMAc/LiCl solvent. Ag particles were synthesized from silver nitrate (Alkaloid, p.a. purity).

### 4.2. Polymerization Reaction

In our previous work [73], the dissolution of cellulose was described. Briefly, cellulose was activated in DMAc at 120 °C for 2 h. The temperature was adjusted to 100 °C and then LiCl (6.6 wt% in DMAc/LiCl solution) was added. The solution was agitated for another hour. After cooling the solution to room temperature, a clear solution containing 5 wt% cellulose was obtained. The polymerization reaction of DMAEMA was carried out in cellulose solution.

The polymerization reaction was carried out in a round bottom flask, in which the cellulose solution was first heated to 90 °C. A solution of DMAEMA and MBA (50 wt%) in DMAc/LiCl was added to the flask after 5 min. Five minutes after the addition of the monomers, a solution of the peroxide initiator in DMAc/LiCl (1 wt% toward monomers) was added to the flask and the reaction was carried out for 3 h [76]. The molar ratio of monomers, crosslinkers, and cellulose for all reaction mixtures is shown in Table S1. The scheme of synthesis and preparation of hydrogels is presented in Supplementary Material Figure S18.

The synthesized polymer was gelled in deionized water or water vapor and formed into spheres or films. For further purification from unreacted monomer and free polymer, the prepared hydrogel samples were kept in deionized water for 5 days and the deionized water was changed every 2 days. After the initial polymerization reaction, part of the reaction mixture was separated and left for analysis. Irradiation of the other part of the mixture was carried out in sealed flasks flushed with N_2_ to create an oxygen-free environment. Irradiation was performed in the Radiation Chemistry and Dosimetry Laboratory of the Ruđer Bošković Institute using the Co-60 γ-panoramic radiation source. Gamma irradiation was used to graft PDMAEMA onto the cellulose, further enhance the conversion of the polymerization reaction, and achieve complete crosslinking of the hydrogel. The samples were irradiated to doses of 10, 30, and 100 kGy at a dose rate of 19.8 kGy h^−1^. The dose mapping of the irradiation facility was carried out experimentally with ionizing chambers and an ECB dosimetry system, as well as by simulation calculations. The irradiation dose and molar ratio of monomers, crosslinking agents, and cellulose for all reaction mixtures are shown in Supplementary Material Table S2.

### 4.3. Microgelation Examination

After polymerization of DMAEMA and MBA in DMAc/LiCl, the reaction mixtures were diluted 5-fold with DMAc/LiCl and filtered through a sinter funnel (pore diameter: 10–16 µm) to separate any microgel particles formed from the reaction mixtures. The cellulose solution in which the polymerization of DMAEMA and MBA was carried out was also 5-fold diluted with DMAc/LiCl. After dilution, the mixtures were centrifuged at 2400 min-1 (relative centrifugal force 1448) for 10 min. The supernatant solution was decanted and further filtered through a sinter (pore diameter 10–16 µm) to completely remove precipitate particles. The polymers from the supernatant solution were precipitated in deionized water to remove DMAc/LiCl, unreacted monomers, and free polymers. The precipitate obtained after centrifugation was purified from DMAc/LiCl and unreacted monomers with deionized water. A schematic of the separation and purification of the reaction mixture is presented in Supplementary Material Figure S19.

Optical microscopy of prepared cel-DMAEMA solution was carried out on an Olympus BX53M microscope in dark field mode in order to determine the presence of microgel particles.

### 4.4. Drying of the Spheres

The freeze-extraction method was used to dry the prepared spheres. Weighed spheres (0.3 g) were placed in a beaker and cooled to −35 °C in a cryostat. The beaker containing the spheres was kept at −35 °C for 30 min and then cold ethanol (35 mL) was poured into the beaker. The beaker was placed in the freezer and the spheres were kept in ethanol at −18 °C for 48 h, with fresh ethanol added after 24 h. After 48 h, ethanol was decanted and the spheres were dried in a vacuum at 50 °C until constant weight.

### 4.5. Preparation of Hydrogels with Silver Particles

The hydrogel films were immersed in 5 mL of silver nitrate solution (*c* = 10^−2^ mol dm^−3^) and the reaction was carried out for 24 h. To remove unreacted silver nitrate from the hydrogel films, the films were removed from the silver nitrate solution and rinsed for 1 h with deionized water. The spheres were dried at room temperature and after 24 h further dried in a vacuum until constant mass.

### 4.6. Adsorption of Fe^3+^ Ions

The spheres of each prepared hydrogel were immersed in 10 mL of iron (III) chloride solution (*c* = 10^−2^ mol dm^−3^). After 18 h, the spheres were rinsed with deionized water for 1 h to remove the non-adsorbed iron (III) chloride.

### 4.7. Polymer Characterization

Characterization of the prepared sample by infrared spectroscopy was performed with the Perkin–Elmer Spectrum One using the ATR module. Measurements were performed at room temperature in the frequency range 650–4000 cm^−1^ with 4 scans and a resolution of 4 cm^−1^. After purification in deionized water, spheres of the sample were weighed and first dried in a laboratory oven and then in a vacuum (90 °C) over phosphorus pentoxide to determine the equilibrium swelling degree. Reswelling of dried samples in deionized water for 150 min was carried out to determine the reswelling degree [76]. For the swelling/reswelling degree, at least 5 spheres were dried/reswollen and the average result is presented. The swelling degree of all samples was performed in deionized water (pH = 5.5) and calculated according to Equation (1):

(1) $$\alpha =\frac{m_{t}-m_{0}}{m_{0}}\times 100\;\%\;$$

where *m*_0_ is the mass of the dry sample and *m*_t_ is the mass of the swelled sample in time *t*.

Rheological tests were performed using a TA Instruments Discovery HR30 hybrid rheometer equipped with a heating/cooling system. To reduce water evaporation, a solvent trap was used during amplitude and frequency sweep tests. All measurements were carried out at a temperature of 24 ± 0.2 °C. Parallel plate geometry with a diameter of 25 mm was used. Furthermore, on the upper plate, sandpaper was attached and a fixed normal force of 1 N was applied to avoid slipping the sample. On each sample, first, the linear viscoelastic region (LVER) was determined with amplitude sweep tests at a fixed frequency of 1 Hz and a strain (γ) varying from 0.01 to 10%. Subsequently, frequency sweep tests were carried out in the frequency range 0.1–100 rad s^−1^ at strain 0.05%. 

A VEGA 3 TESCAN microscope with a detector of secondary electrons was used for scanning electron microscopy (SEM). Additionally, energy dispersive X-ray spectrometry (EDS) was carried out in order to analyze the chemical composition of samples. All samples were previously sputter coated with a Au/Pd alloy in argon plasma to enhance their electrical conductivity. Due to the resolution constraints of the instrument, the size and morphology of the silver particles could not be determined. 

The number average molecular weight between crosslinks, *M*_c_, was estimated by the classical rubber elasticity theory using Equation (2) [77]:

(2) $$G^{\prime }=\frac{\rho \;R\;T}{M_{c}}\;\;\;\;\;\;\;$$

where *G*’ is the shear modulus, ρ is the density of the polymer in the network and it is equal to the dry mass divided by the hydrogel volume, *R* is the gas constant, *T* is absolute temperature, and *M*_c_ is the number average molecular weight between crosslinks.

Taking into account the free ends and the loop of the polymer network strands, Equation (2) is adjusted to Equation (3) [77]:

(3) $$G^{\prime }=\frac{\rho \;R\;T}{M_{c}}\;\left(1-\frac{2M_{c}}{M}\right)\;\;\;\;\;\;$$

where *M* is the initial molecular weight of the polymer.

## Figures and Tables

**Table 1:** Content of nitrogen in prepared cellulose-based hydrogels determined by SEM-EDS.

Sample	*w* (*N*) (wt%)	±*σ* (wt%)
1-1 CP	n. d. *	n. d. *
1-1 100	1.8	0.1
1-3 CP	3.1	0.2
1-3 100	5.9	0.4
1-5 CP	4.8	0.2
1-5 100	7.9	0.3

* n. d.—not determined.

**Table 2:** Molecular weight between crosslinks for prepared hydrogel samples.

Sample	*M*_c_ (g mol^−1^)
Cellulose	5373
1-1 CP	5239
1-1 100	7165
1-3 CP	9411
1-3 100	9431
1-5 CP	10231
1-5 100	7945

**Table 3:** The content of the silver in the prepared hydrogels was determined by SEM-EDS analysis.

Sample	*w* (Ag) (wt%)	±*σ* (wt%)
Cellulose	0.9	0.2
1-1 CP	1.2	0.2
1-1 100	1.8	0.2
1-3 CP	4.0	0.3
1-3 100	3.9	0.5
1-5 CP	3.1	0.3
1-5 100	5.5	0.3

## Data Availability

Data is contained within the article or supplementary material.

## Supplementary Materials

The following supporting information can be downloaded at https://www.mdpi.com/article/10.3390/gels9020094/s1, Figures S1 and S2: Reaction mixtures after polymerization; Figures S3–S5: Optical micrographs of microgel particles, Figure S6: Infrared spectra of hydrogels irradiated with different doses, Figures S7–S14: Infrared spectra of separated microgel particles (samples with abbreviation P) and the polymer obtained from supernatant solution (abbreviation F), Figures S15–S17: Prepared samples of hydrogels before and after the reaction with silver nitrate, Figure S18: Synthesis and hydrogel preparation methods, Figure S19: Separation of formed components after polymerization reaction in cellulose solution, Table S1: Weight percentage of PDMAEMA microgel particles in the reaction mixture, Table S2: Molar ratio of reactants (monomer (DMAEMA), crosslinking agent (MBA), and cellulose (cel)), and irradiation dose for prepared samples, Table S3: Table S3 Values for the critical strain of prepared hydrogels.
