# Supplementary material for: Swelling and Viscoelastic Properties of Cellulose-Based Hydrogels Prepared by Free Radical Polymerization of Dimethylaminoethyl Methacrylate in Cellulose Solution
Source: Gels. 2023 Jan 21;9(2):94. doi: 10.3390/gels9020094 (PMC9956197; doi:10.3390/gels9020094)
Supplement: Supplementary file 1 [file gels-09-00094-s001.zip › gels-2098148-supplementary.pdf]

Swelling and viscoelastic properties of cellulose based hydrogels prepared by free radical polymerization of dimethylaminoethyl methacrylate in cellulose solution

Roko Blažic[1], Katarina Marušić[2], Elvira Vidović[1]

[1]*University of Zagreb, Faculty of Chemical Engineering and Technology,*

Maruličev trg 19, HR-10000 Zagreb, Croatia

[2]*Ruđer Bošković Institute, Division of Materials Chemistry, Radiation Chemistry and Dosimetry Laboratory,*

Bijenička cesta 54, HR-10000, Zagreb, Croatia

Supplementary material

*Correspondence to:

E-mail address: evidov@fkit.hr (Elvira Vidović), Marulićev trg 19, 10000 Zagreb, Croatia

Table S1 Molar ratio of reactants (monomer (DMAEMA), crosslinking agent (MBA) and cellulose (cel)) and irradiation dose for prepared samples

Sample	*n*(cel)/*n*(DMAEMA)	*n*(DMAEMA)/*n*(MBA)	Irradiation dose (kGy)
1-1 CP	1:1	15:1	0
1-1-10			10
1-1-30			30
1-1-100			100
1-3 CP	1:3	15:1	0
1-3-10			10
1-3-30			30
1-3-100			100
1-5 CP	1:5	15:1	0
1-5-10			10
1-5-30			30
1-5-100			100

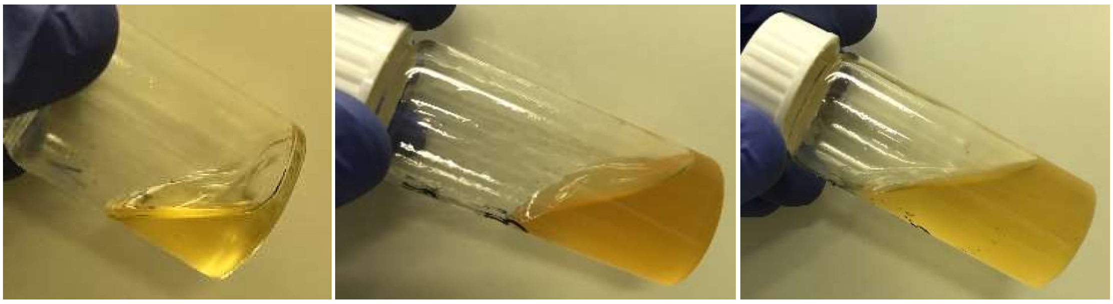

Figure S1. Reaction mixtures after polymerization a) sample 1-1 CP, b) 1-3 CP, c) 1-5 CP.

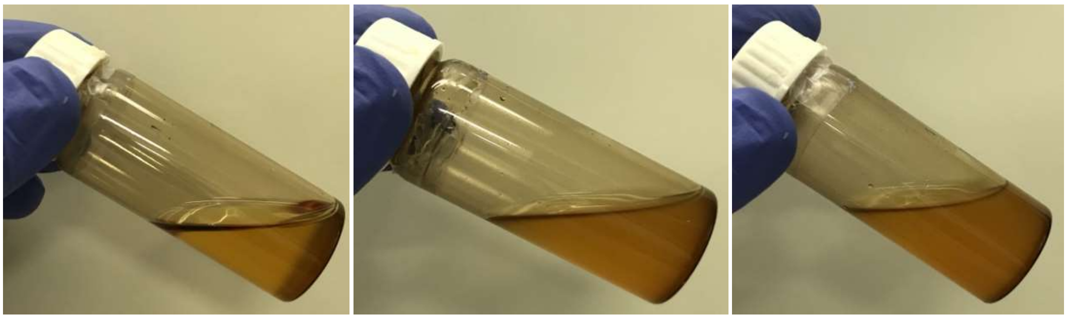

Figure S2. Reaction mixtures after irradiation with a dose of 100 kGy a) sample 1-1 100, b) 1-3 100, c) 1-5 100.

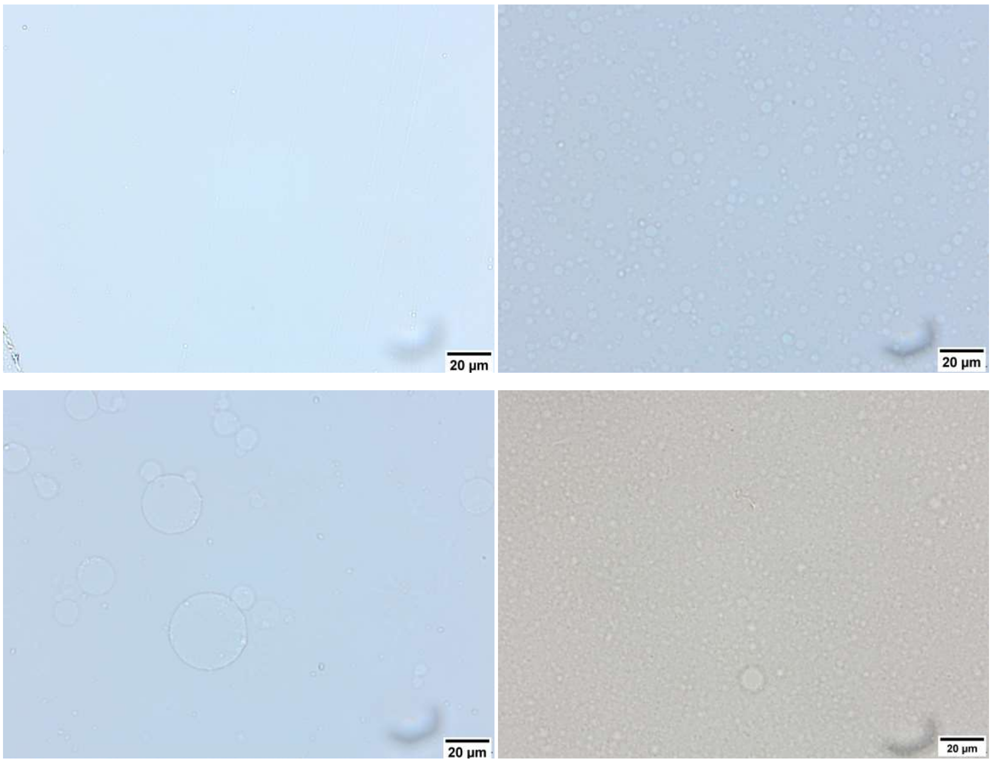

Figure S3. Optical micrographs for reaction mixture of 1-1 CP, 10, 30, 100 samples.

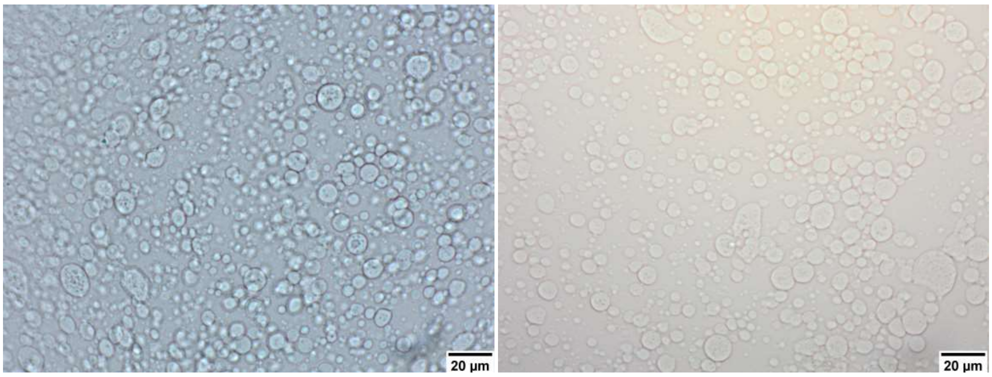

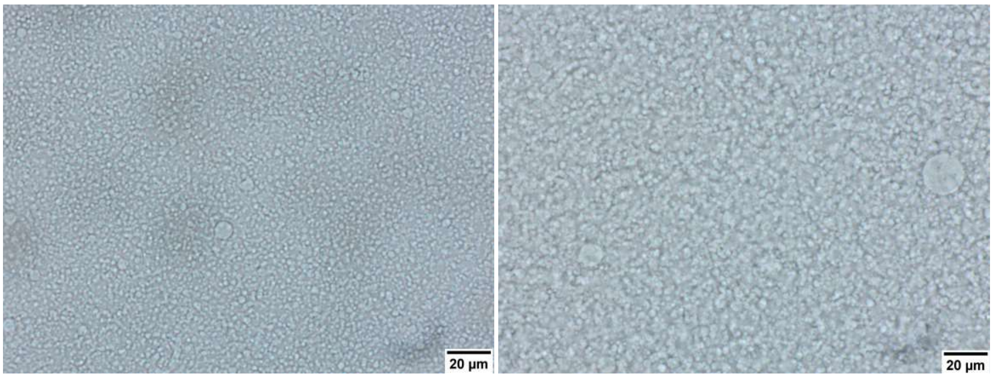

Figure S4. Optical micrographs for reaction mixture of 1-3 CP, 10, 30, 100 samples.

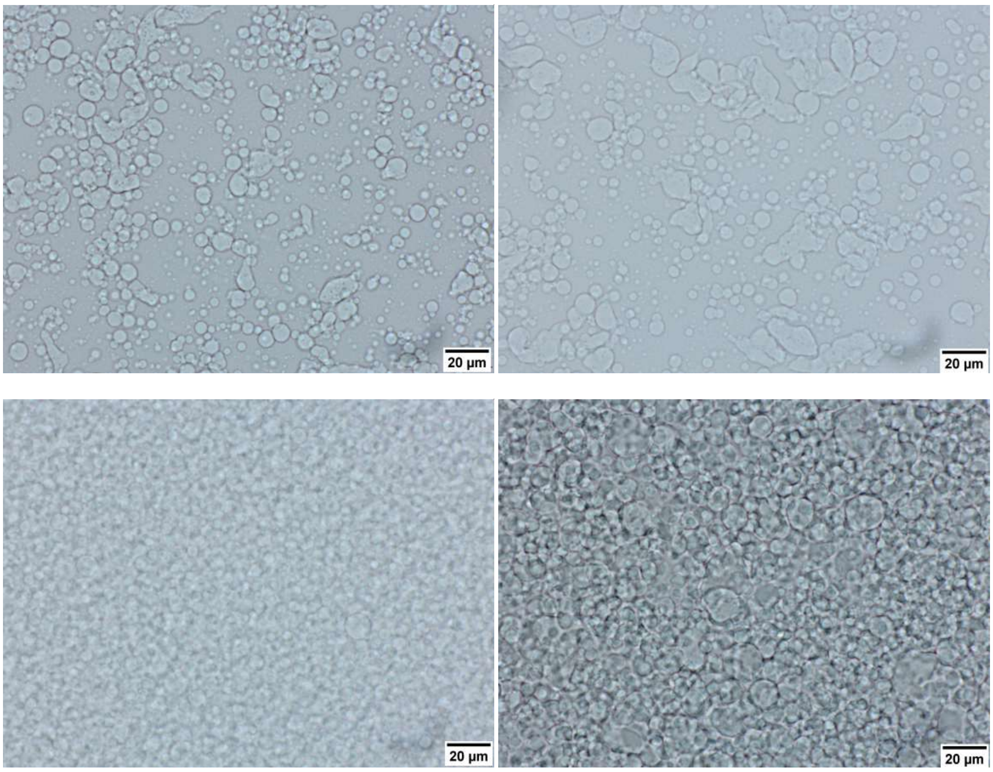

Figure S5. Optical micrographs for reaction mixture of 1-5 CP, 10, 30, 100 samples.

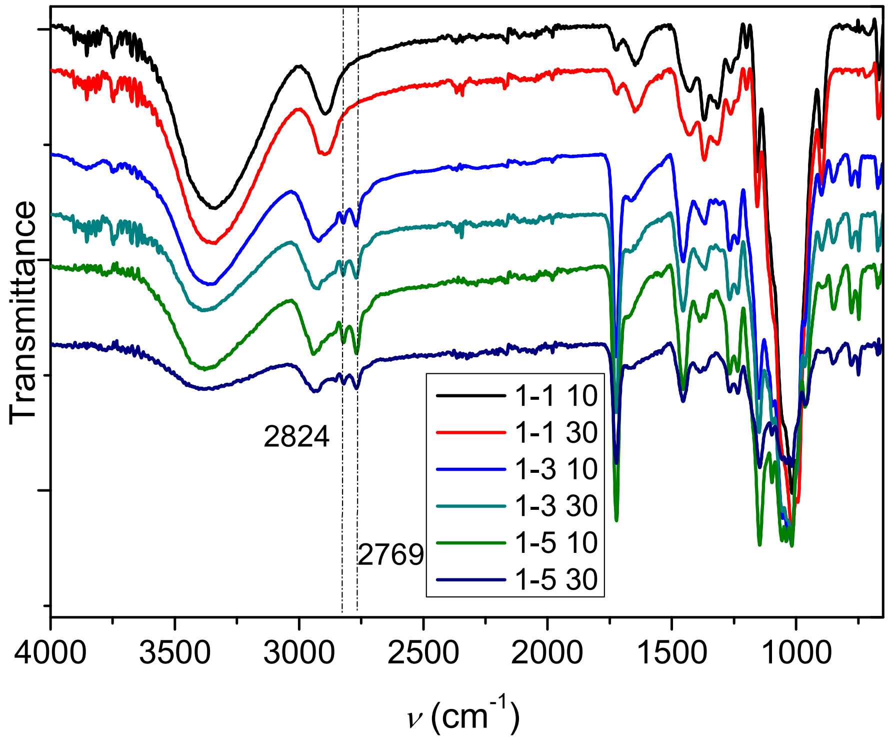

Figure S6. Infrared spectra of hydrogels 1-1, 1-3 and 1-5 irradiated with different doses (10, 30, 100 kGy).

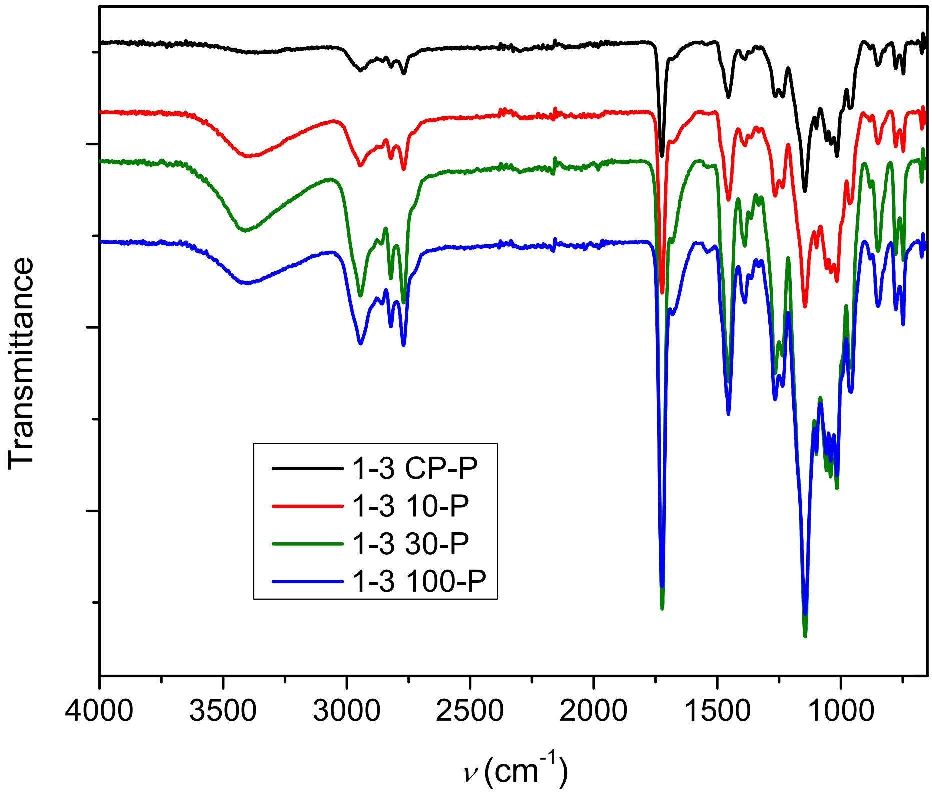

Figure S7 Infrared spectra of samples obtained from precipitate after centrifugation (abbreviation P).

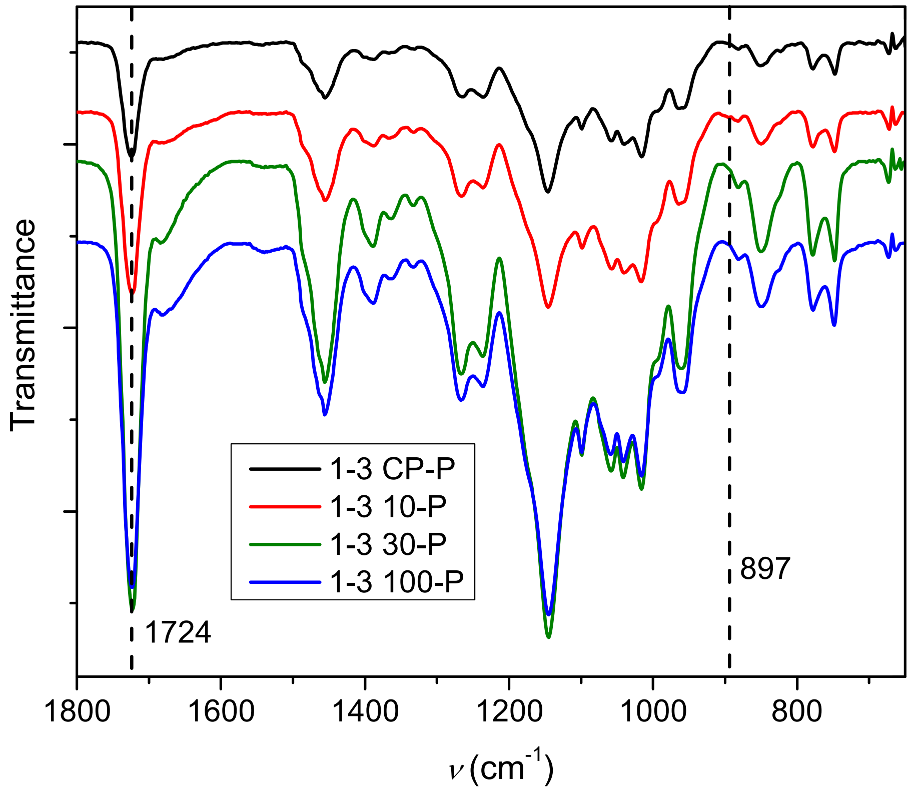

Figure S8. Infrared spectra of samples obtained from precipitate after centrifugation (abbreviation P) (magnified spectra).

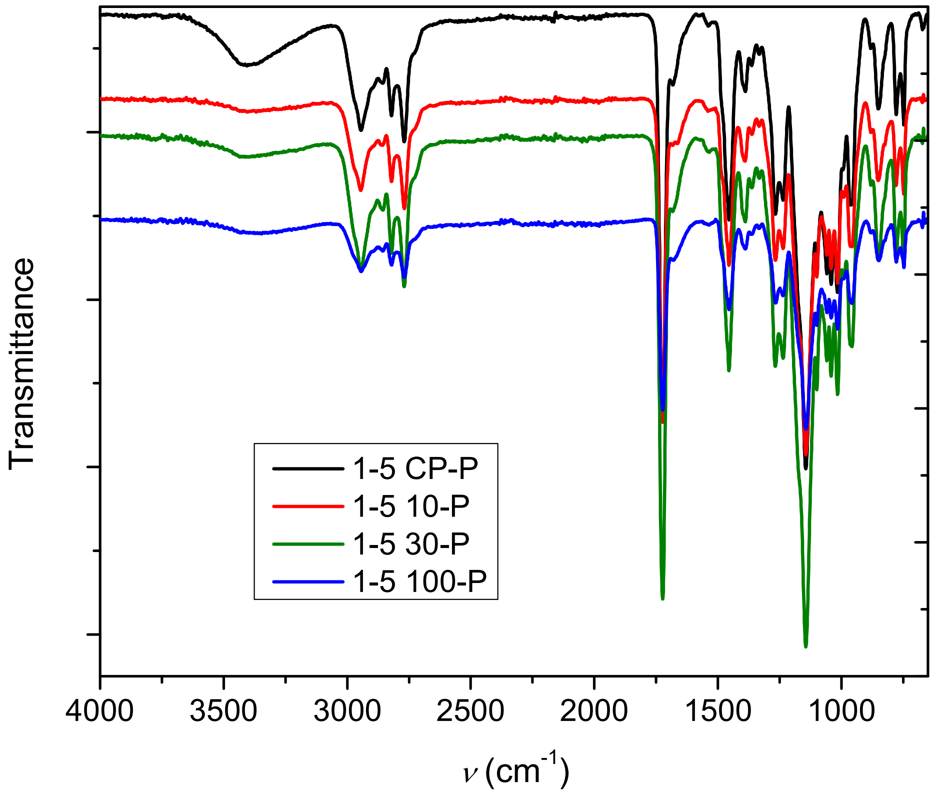

Figure S9. Infrared spectra of samples obtained from precipitate after centrifugation (abbreviation P) .

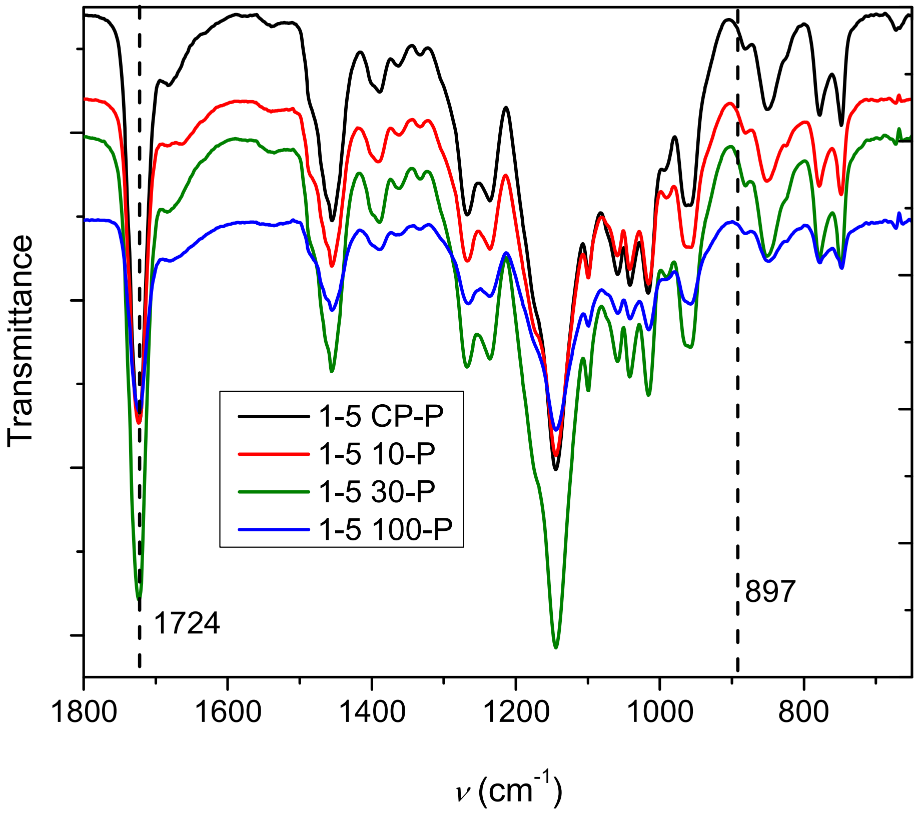

Figure S10. Infrared spectra of samples obtained from precipitate after centrifugation (abbreviation P) (magnified spectra).

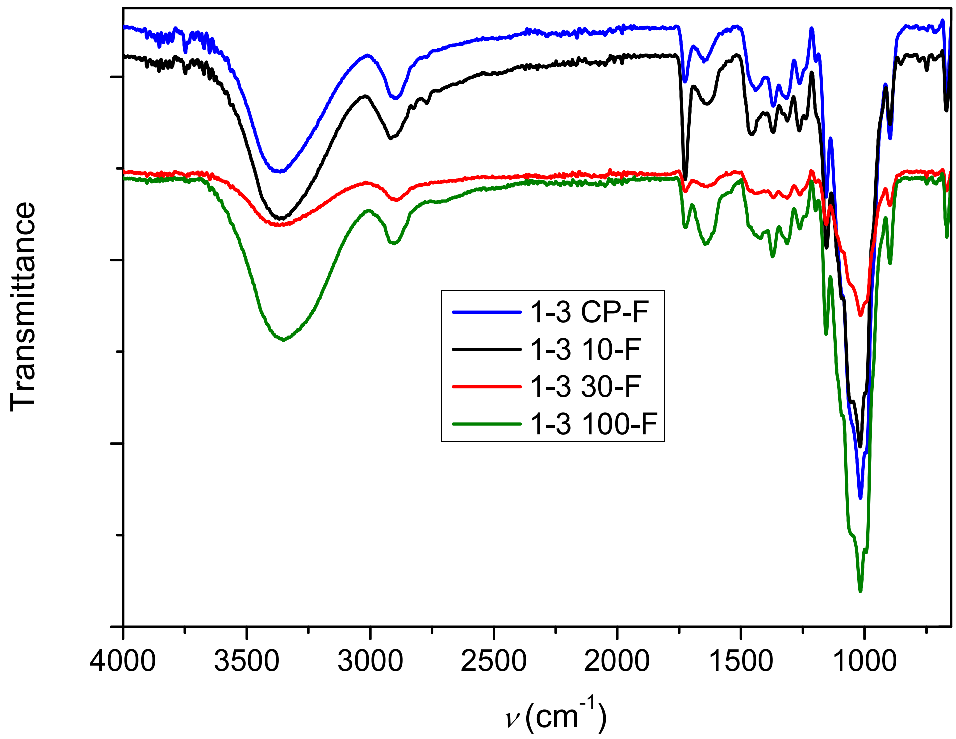

Figure S11. Infrared spectra of polymer precipitated from supernatant solution of 1-3 samples .

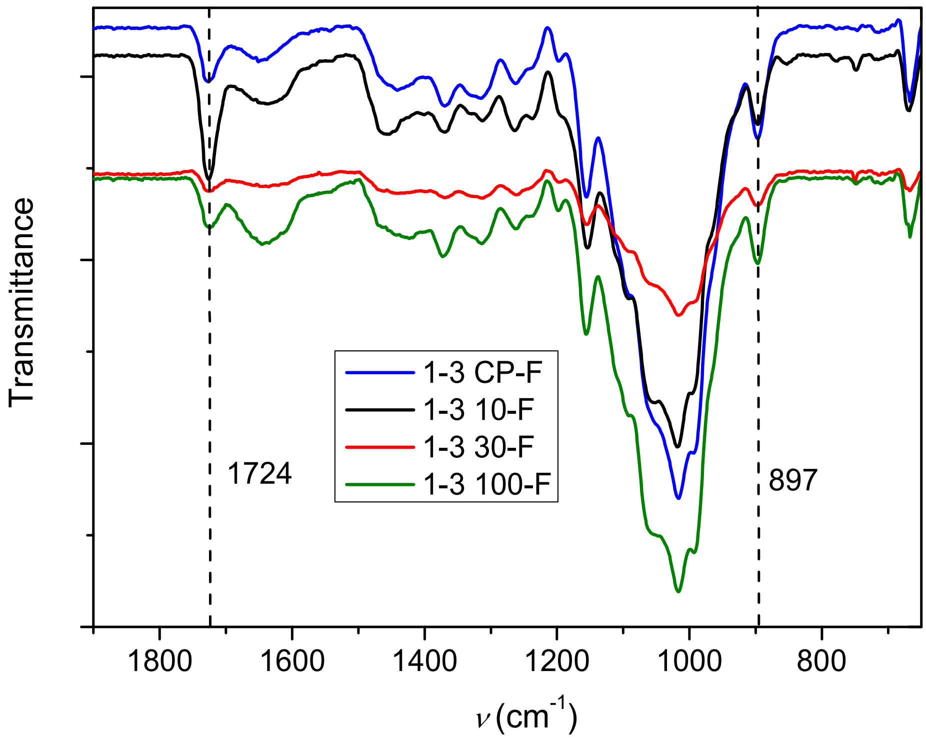

Figure S12. Infrared spectra of polymer precipitated from supernatant solution of 1-3 samples (magnified).

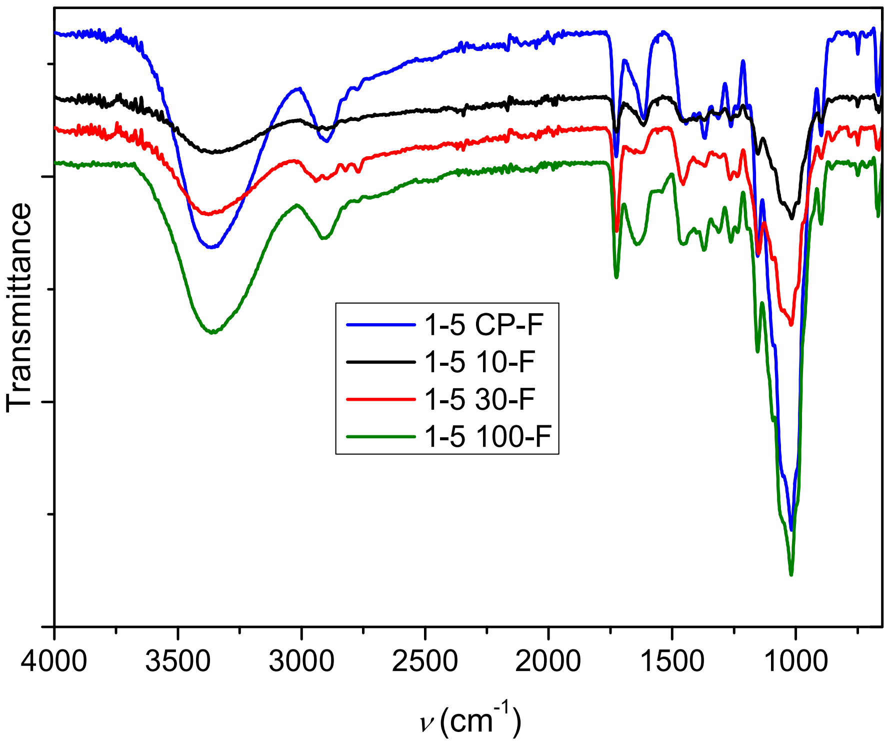

Figure. S13. Infrared spectra of polymer precipitated from supernatant solution of 1-5 samples.

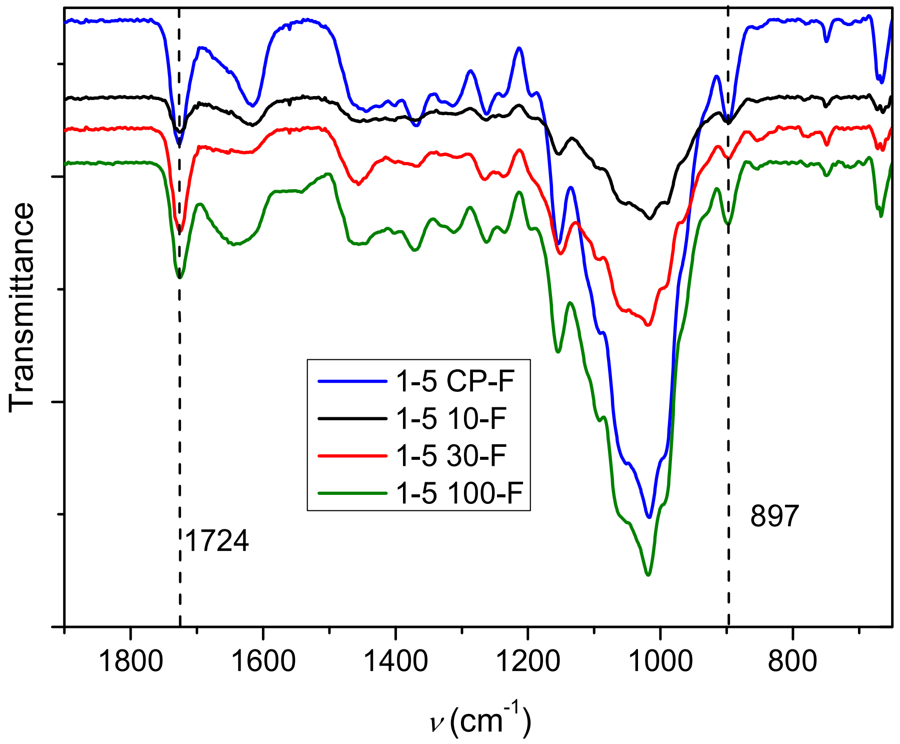

Figure S14. Infrared spectra of polymer precipitated from supernatant solution of 1-5 samples (magnified).

Table S2 Weight percentage of PDMAEMA microgel particles in reaction mixture

Sample	*w*(PDMAEMA microgel) / %
1-3 CP-P	0.6
1-3 10-P	1.1
1-3 30-P	2.1
1-3 100-P	4.1
1-5 CP-P	5.0
1-5 10-P	5.3
1-5 30-P	8.4
1-5 100-P	10.5

Table S3 Values for critical strain of prepared hydrogels

Sample	$\log(\gamma_s)$
Cellulose	0.6
1-1 CP	0.9
1-1 100	1.2
1-3 CP	1.2
1-3 100	1.4
1-5 CP	1.0
1-5 100	1.2

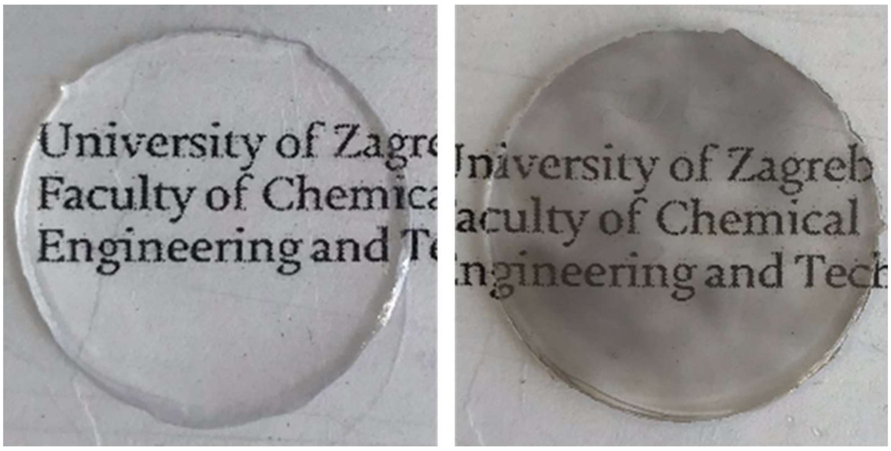

Figure S15. Prepared samples of neat cellulose before reaction with silver nitrate (left) and after reaction with silver nitrate (right).

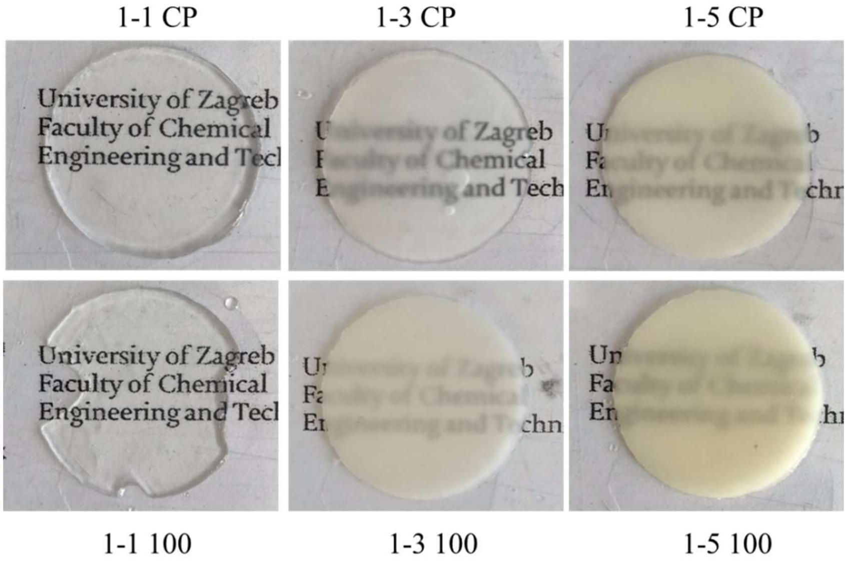

Figure S16. Prepared samples of hydrogels before reaction with silver nitrate.

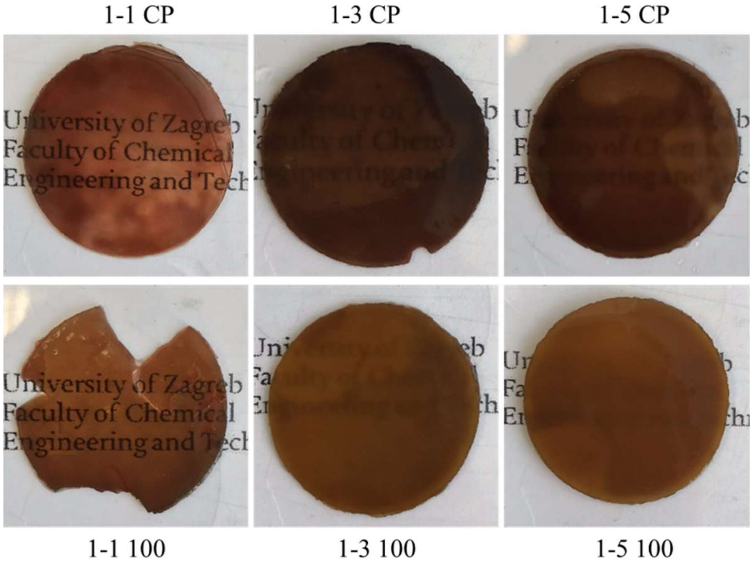

Figure S17 Prepared samples of hydrogels after reaction with silver nitrate

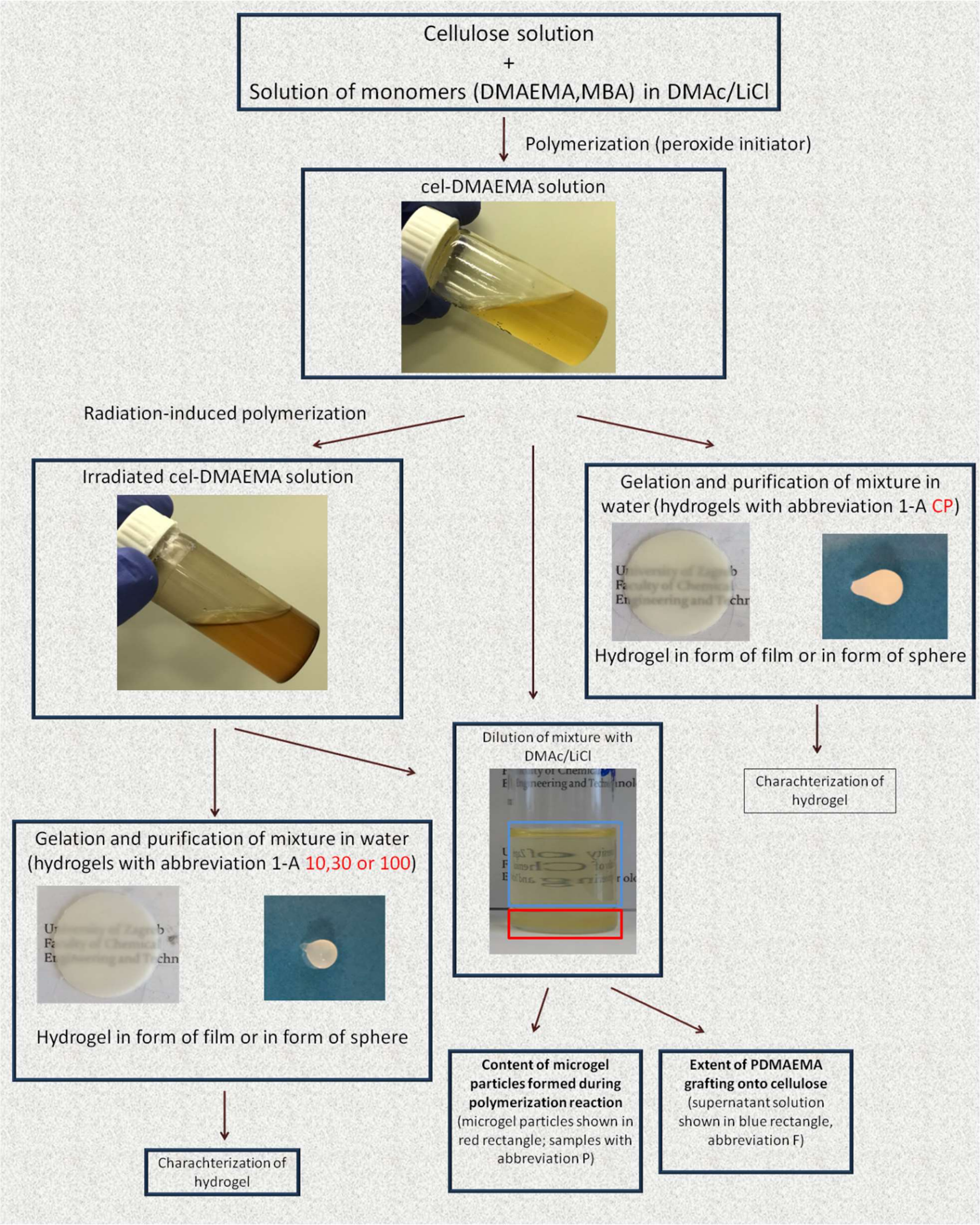

Figure S18. Synthesis and hydrogel preparation method.

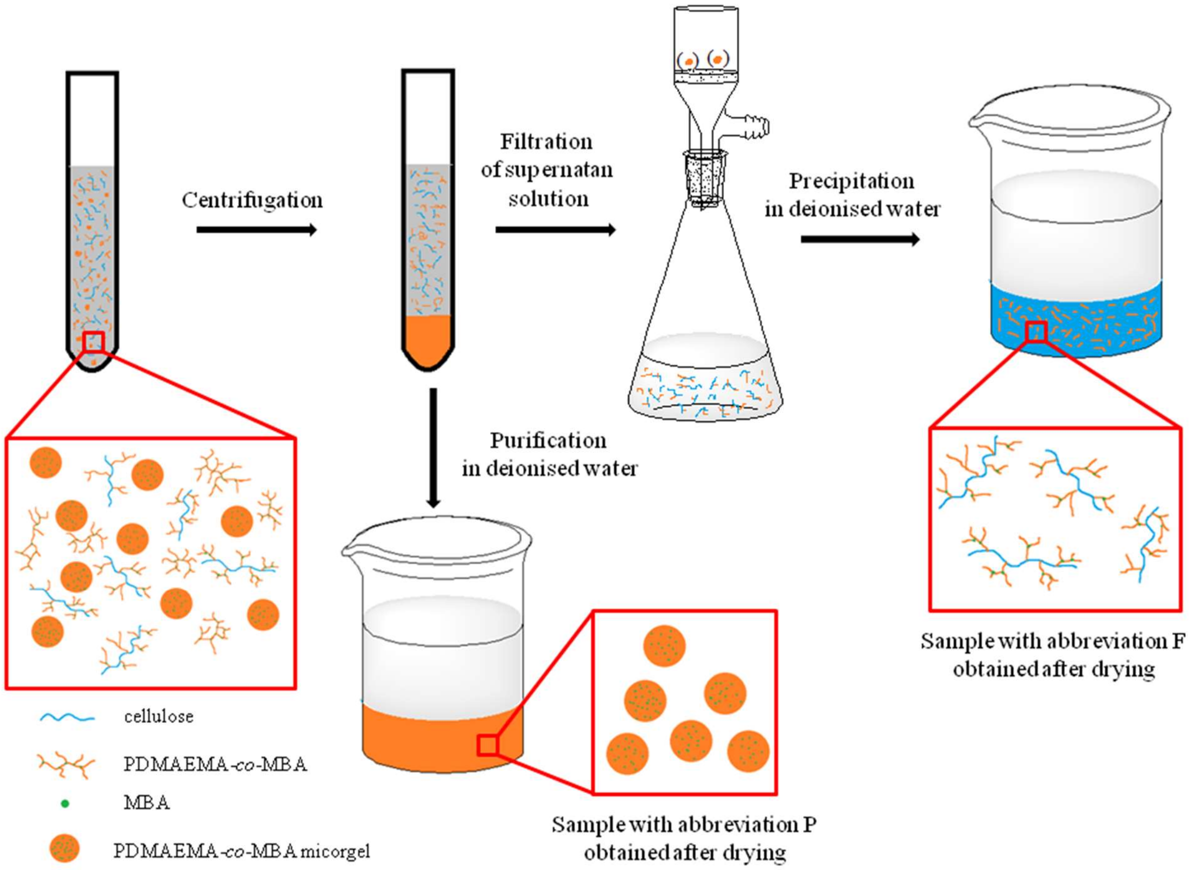

Figure S19. Separation of formed components after polymerization reaction in cellulose solution.
